# Sustainable water remediation with algae-derived adsorbents: from synthesis strategies to adsorption performance and mechanisms

**DOI:** 10.1039/d5ra09740a

**Published:** 2026-04-08

**Authors:** Tongtong Wang, Soni Patyal, Mu. Naushad, Pooja Dhiman, Gaurav Sharma, Ying Gao, Sen Wang, Weiqian Li, Jinjun Cai

**Affiliations:** a Institute for Interdisciplinary and Innovation Research, Xi'an University of Architecture and Technology Xi'an 710055 P.R. China tongtwang@163.com; b International Research Centre of Nanotechnology for Himalayan Sustainability (IRCNHS), Shoolini University Solan 173229 India gaurav.541@shooliniuniversity.com; c Department of Chemistry, College of Science, King Saud University P.O. Box 2455 Riyadh 11451 Saudi Arabia; d School of Resources Engineering, Xi'an University of Architecture and Technology Xi'an 710055 P. R. China; e Institute of Agricultural Resources and Environment, Ningxia Academy of Agriculture and Forestry Sciences Yinchuan 750002 P.R. China; f The Crop Research Institute, Ningxia Academy of Agriculture and Forestry Science/Ningxia Crop Breeding Engineering and Technology Research Center Yinchuan 750002 P. R. China nxyccai@163.com

## Abstract

Human-driven activities related to diverse industries such as textiles, pharmaceuticals, plastics, leather, and agriculture contribute significantly to the discharge of pollutants into aquatic environments, thereby threatening the ecological balance and posing a risk to living organisms. Over the past few years, algae have been acknowledged as a cost-effective and sustainable resource for the detoxification of harmful pollutants, primarily through mechanisms such as intracellular biodegradation, bioaccumulation, and biosorption. Besides the direct involvement of algae in the removal of pollutants, they can be converted into carbon-rich materials such as hydrochar, biochar, and activated carbon. These materials possess high specific surface areas and different functional groups, which make them quite effective for the adsorption of organic pollutants in wastewater treatment. Algal-derived adsorbents exhibit high adsorption efficiency because of the synergistic effects of various interactions, including electrostatic forces, hydrogen bonding, π–π interactions, and pore filling effects, all of which depend on the engineered surface functional groups and porous structure of algae-derived carbon-rich materials. This review uniquely explores various algal species for the preparation of adsorbents and also examines the modification methods used to convert algae into adsorbents. It examines their effectiveness in the removal of organic contaminants from water systems. Future research needs to bridge the gap between laboratory-scale and real-world applications, especially through pilot-scale studies in real wastewater and comprehensive life-cycle assessments.

## Abstract 2

This review covers the synthesis, modification, and mechanisms of algae-derived carbon-rich adsorbents for sustainable removal of human-driven industrial pollutants from water, noting their high efficiency, reusability, and application potential.

## Graphical abstract

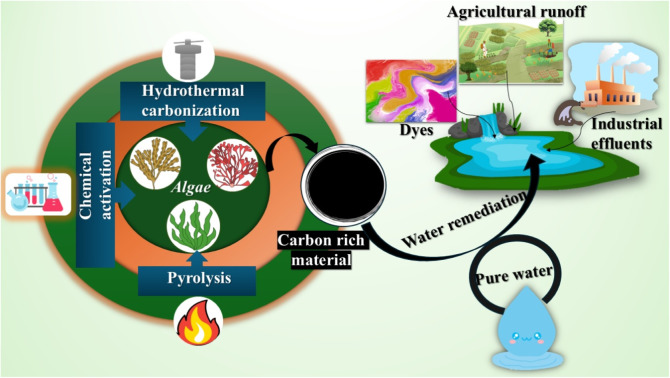

## 1. Introduction

The growth of industries and human activities have been the main reasons for pollutant accumulation in water, including dyes, pharmaceuticals, pesticides, herbicides, and polyaromatic hydrocarbons, which are very harmful not only to human health but also to aquatic life. Therefore, obtaining clean and safe water remains the biggest challenge, mostly in less developed areas, where local communities are more affected by water borne diseases and the environmental problems.^1^ A large amount of organic pollutants has been detected in water bodies, thus becoming a major cause of concern for the environment and human health because of their toxic effects and the risk of cancer development posed by them. In particular, these pollutants consist of antibiotics, hydrocarbons, phenols, dyes, industrial additives, and agrochemicals. Organic pollutants are a group of harmful substances that have the potential to damage biological structures even at the cellular level. Their stable chemical constituents can affect cells, organs, tissues and disrupt microorganisms in the soil ecosystem.^2^ Their presence has been associated with a variety of both acute and chronic health disorders in humans, and they may pose risks to fetal development. In view of the hazardous nature of these compounds, their controlled use and eventual removal from the wastewater have become major concerns. As a result, numerous treatment methods have been developed to ensure that these pollutants are removed effectively from contaminated water. Conventional methods for treating wastewater, such as membrane filtration, ion exchange, reverse osmosis, chemical precipitation, biological oxidation, and electrochemical techniques, have been widely used for pollutant removal. However, their high operational costs, high energy consumption, and large space requirements limit their effectiveness; thus, it is necessary to develop more sustainable and efficient alternatives.^3^

Various in-depth research works have been conducted, mainly focusing on the identification, refinement, and engineering of high-performance sorbents that effectively remove pollutants from aqueous environments. Among the wide range of materials tested, algae have been recognized as the most promising because of their dual capacity to absorb CO_2_ and purify wastewater, along with algal uptake and biomass production, delivering carbon-rich substances that make it possible to close the material cycle in a sustainable and green way from aquatic systems.^4,5^ Carbon-rich materials derived from algae such as activated carbon, hydrochar, and biochar obtained from pyrolysis, hydrothermal carbonization, and torrefaction, respectively, have high adsorption efficiency for wastewater remediation due to their porous structure, aromatic stability, large surface area, and numerous functional groups, which allow pollutants to be removed effectively through enhanced cation exchange and surface interaction mechanisms, hence emerging as promising materials for environmental remediation.^6,7^

To write this review, we searched the relevant literature in databases (or websites) such as Web of Science, Scopus, Google Scholar, PubMed, *etc.*, and the search keywords included, but were not limited to, algae, algae-derived biochar/carbon, adsorbent, water treatment/purification, organic pollutants, pharmaceutical wastewater, removal/adsorption, modification, synthesis, adsorption mechanism, and searched by keyword combinations to ensure coverage and representativeness. To encompass the most current developments in synthesis techniques, adsorption characteristics, and the mechanism of algal-based adsorbents, the literature section concentrated on high quality studies published within the last ten years. With an emphasis on the elimination of artificial dyes and pharmaceutical residues, this article offers a comprehensive analysis of carbon-rich materials derived from algae for water purification.

The focus is on using microwave assistance, chemical functionalization, and pyrolysis to produce these materials. The physical and chemical properties of these materials are critically evaluated. Moreover, the discussion goes into great depth on how these materials work incredibly well to adsorb organic pollutants after surface modification. Furthermore, the research discussed herein considers the regeneration and reuse processes of adsorbents, highlighting their technological viability in environmentally friendly or sustainable water treatment systems.

## 2. Organic contaminants in the water system

This section focuses on the various types of organic pollutants that have become a significant concern, such as pharmaceutical residues, personal care products, agrochemicals (pesticides and herbicides), synthetic dyes (methylene blue, methyl orange, crystal violet, and Congo red), phenolic compounds and hydrocarbons. Most of these compounds are characterized by aromatic and planar molecular frameworks, which may consist of ring-based polymeric structures.^8^ These structures provide the compounds with very high chemical stability, and consequently these compounds persist for a long time in the environment and are very difficult to degrade naturally.

### 2.1 Synthetic dyes

Synthetic dyes are artificial chemically synthesized colorants, which are generally used in industries such as cosmetics, food processing, pharmaceuticals, textiles, fuel marking, and leather.^9^ Among these sectors, the textile industry is the main cause of dye pollution in water bodies. The processes of dyeing show fixation losses that range from 5–50% depending on the dye–fabric interactions, which result in the release of coloured effluents of about 200 billion litres annually into aquatic environments without treatment.^10–12^ Every year, about 4.5 million tons of azo dyes, along with their degradation products, many of which are mutagenic, ecotoxic, and carcinogenic, are dumped in water bodies, where they change nutrient dynamics, lower the oxygen levels, kill organisms, and hence indirectly introduce risks to human health through biomagnification and bioaccumulation along the food chain.^13–15^

### 2.2 Agrochemicals

Agrochemicals are defined as chemical substances, either man-made or natural, that are used in agriculture to increase yield and take care of the health of plants. They include fertilizers, pesticides (such as herbicides and insecticides), and growth-regulating compounds that can influence the physiological processes in plants.^16^ Modern farming relies heavily on agro-pesticides to keep pests, weeds, and diseases under control; however, their persistence in the soil and crops is causing worries about their leaching into water bodies and build-up in the food chain.^17^ Over 2 million tonnes of pesticides are used annually worldwide, with China being the largest user, followed by the United States and Argentina, both showing a sharp increase in application rates.^18^ The global fertilizer and pesticide application rates increased from 87.25 to 118.62 kg ha^−1^ and 1.47 to 2.26 kg ha^−1^, between 2001 and 2021, respectively, demonstrating the increasing use of agrochemical across farming systems; however, regional variations in usage levels persist.^19^ Although agrochemicals are meant to improve soil fertility and prevent plant diseases, their extensive use can damage the soil structure, disturb natural microbial ecosystems, leave harmful residues on crops, and have serious negative effects on human health, including genotoxic, mutagenic, and carcinogenic effects.^20,21^

### 2.3 Pharmaceuticals and personal care products

The main sources of pharmaceutical pollutants in natural bodies are sewage systems that contain medical leftovers from human excrement, healthcare facilities, and effluents from drug manufacturing industries.^22^ Conversely, many chemicals are released from personal care items, such as shampoos, sunscreen, insect repellents, toothpaste, soaps, facewash, and perfumes. Because siloxanes and amine-based compounds such as monoethanolamine are commonly found in hair dyes and detergents, and the daily use of shower gel and shampoo alone can release approximately 9574 µg and 49.25 µg per person per day, respectively.^23^ This illustrates how routine hygiene practices contribute to chemical emissions. Because of their complex chemistry, poor detectability, and limited toxicity research, pharmaceuticals and personal care products, including veterinary medications, frequently avoid wastewater treatment, pollute ecosystems, and provide unrecognized risks.^24,25^

### 2.4 Phenolic compounds and hydrocarbons

Aquatic ecosystems are persistently threatened by the high concentrations of phenolic pollutants found in industrial wastewater, which can reach up to 1200 mg L^−1^ from coke ovens, 1220 mg L^−1^ from petrochemicals, 6800 mg L^−1^ from coal mining, and 500 mg L^−1^ from oil refineries, while other industries that release these pollutants include textiles, pharmaceuticals, leather, and paper, which can reach up to 1600 mg L^−1^.^26,27^ Also, coal gasification processes escalate the situation of environmental risks even more as they lead to the release of ammonia, phenols, thiocyanate, and persistent polycyclic aromatic hydrocarbons that resist degradation, bioaccumulate in living tissues, and cause teratogenic, mutagenic, and carcinogenic effects in ecosystems and in humans.^27–29^ Additionally, the chemical, physical, and biological integrity of the soil is compromised by hydrocarbon pollution from petroleum refineries, which results in decreased enzyme activity, nutrient depletion, and poor water and oxygen availability.^30^ These changes disrupt the soil retention, reduce agricultural productivity, and impair plant growth, ultimately causing erosion and surface water pollution.^30,31^

### 2.5 Health risks from organic pollutant exposure

**Fig. 1:**
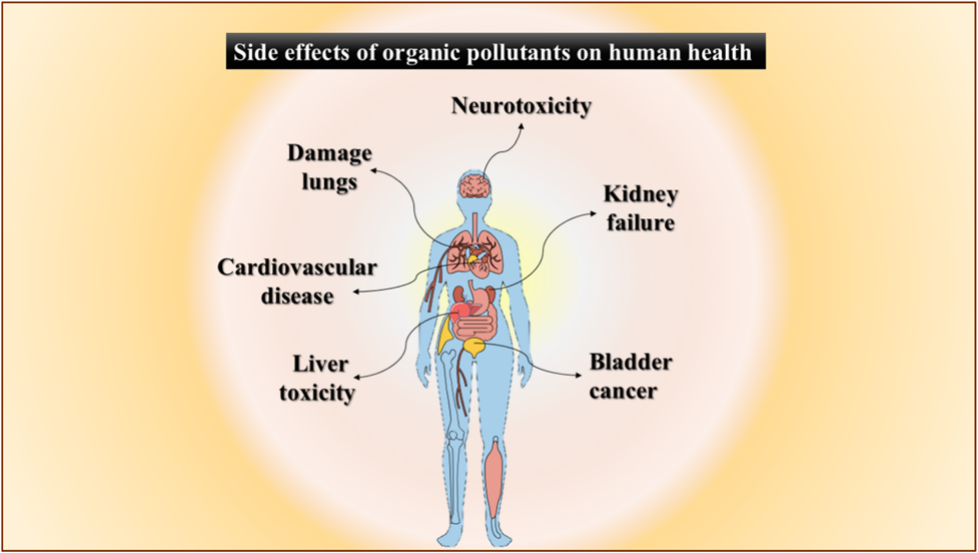
Visual diagram illustrating the toxic impacts of organic pollutants on human health.

Agricultural and industrial pollutants such as dyes, hydrocarbons, pesticides, petroleum derivatives, and nitrogenous compounds pose long-term environmental risks due to their toxicity, mobility, and resistance to natural degradation.^32,33^ Organic pollutants such as detergents, industrial solvents, plastic additives, and pesticide residues infiltrate soil and water, leading to reproductive dysfunction, immunosuppression, cardiovascular disorders, and acute illnesses such as nausea, diarrhea, and skin irritation with heightened risks from chronic exposure and bioaccumulations (Fig. 1) in contaminated environments.^34^ Tetracycline, a widely used antibiotic, causes gastrointestinal disturbances, hepatoxicity, photosensitivity, hypersensitivity, risk of pseudotumor cerebri, developmental effects on pediatric teeth and bones, and *Clostridioides difficile*-associated diarrhea.^35^ Malachite green, a widely used synthetic dye in aquaculture and industry, exhibits environmental persistence and induces teratogenic, carcinogenic, and reproductive toxicity, promoting regulatory bans in developed nations such as Canada, the United States, and countries across the European Union.^36–40^ Congo red (CR), another synthetic dye, poses mutagenic, cytotoxic, and carcinogenic risks to humans and induces enzyme inhibition, while its metabolites disrupt aquatic species through genotoxicity and biochemical interference.^41^

### 2.6 Various methods for removing organic pollutants from wastewater

Organic pollutants are persistent and structurally stable in environmental systems because of their complex aromatic and sterically hindered molecular structure. Numerous techniques, including filtration, coagulation, distillation, crystallization, extraction, oxidation, precipitation, membrane processes, and adsorption, can be used to eliminate these contaminants from water.^42^ However, one or a combination of methods is selected based on the criteria of operational effectiveness, environmental compatibility, and economic feasibility.^43^ Even though there are many different water treatment methods, each one has unique benefits and disadvantages that are influenced by factors such as economic viability, pollutant concentration, energy requirements, scalability, and overall environmental sustainability. Here, we present a summary of the challenges associated with all these methods in Table 1. This comparison draws attention to why adsorption is still widely explored despite its operational limitations.

**Table 1:** Various water purification methods: advantages and challenges

Methods for water purification	Advantages	Challenges	Ref.
Filtration: filtration refers to the action of filtering unwanted contaminants from water to make it clean and fit for consumption	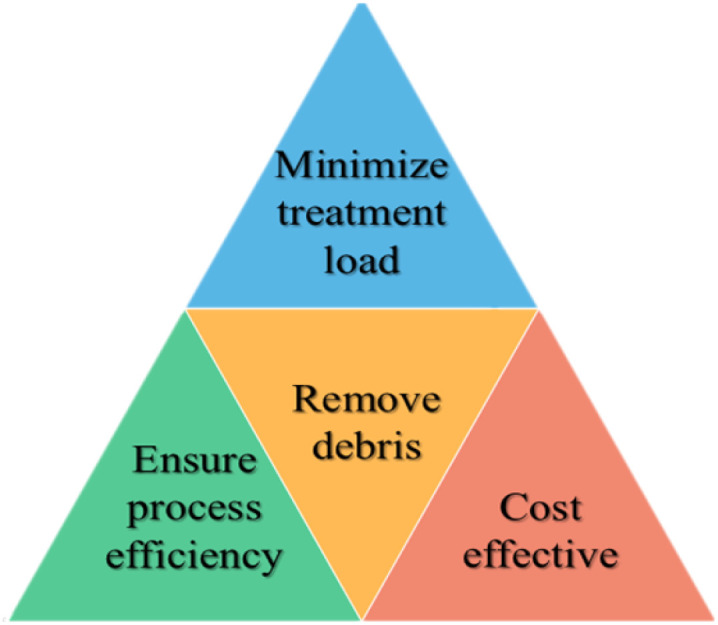	Poor removal of dissolved and fine pollutants, limited efficiency for organic contaminants, needs maintenance to prevent clogging, and produces sludge, which requires proper disposal	44 and 45
Coagulation: in water treatment, this is a chemical process of aggregating fine particles into larger, settleable flocs	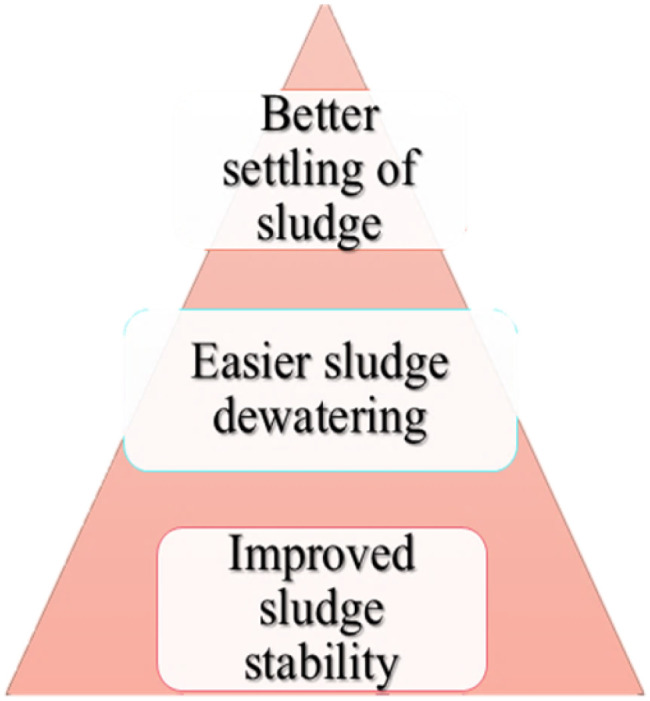	Coagulation requires delicate pH management with alkaline additives, but is still complicated, generates hazardous sludge, is less efficient, and is dependent on proper functioning, which limits its reliability in regular water purification	46–48
Distillation: distillation is a separation process that is used to separate the components of a mixture based on their relative volatility	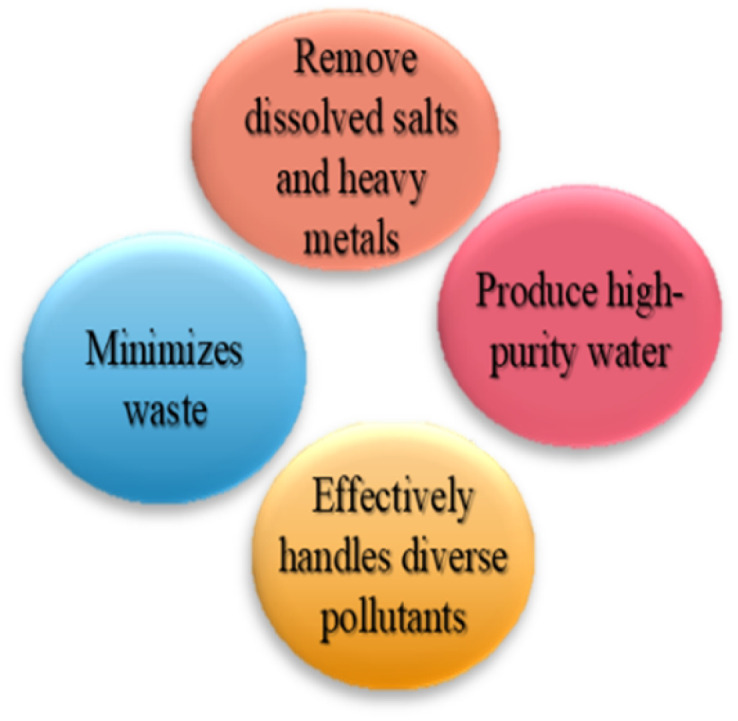	Distillation separates impurities by changing their phases, but it requires significant energy. Besides, volatile compounds are not removed, hence its efficiency is limited	46
Crystallization: it is a process of separating solids from liquids during which the dissolved solute comes out of the solution as pure crystalline solids	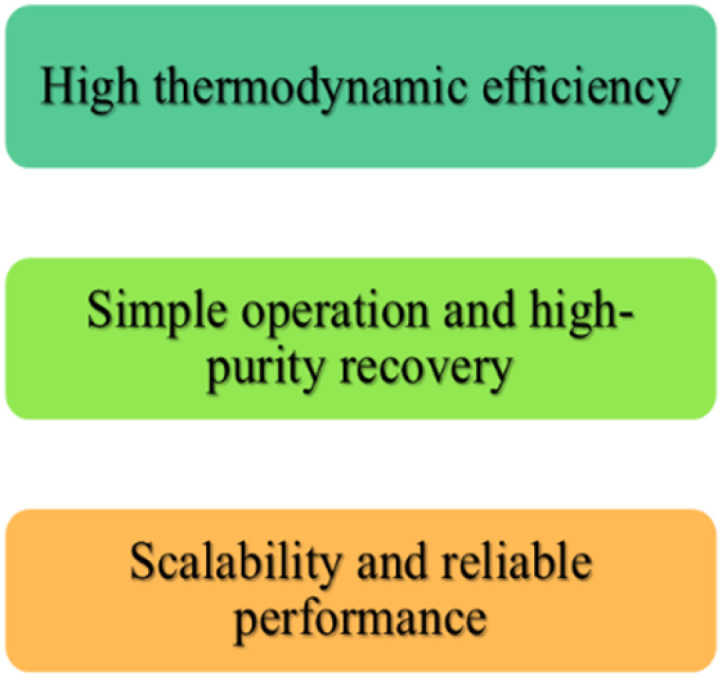	The crystallization process has several problems, such as high capital and energy consumption, mechanical complexity, dependence on agents or heating, operational difficulties, limited applicability, and restricted treatment capacity	49
Oxidation: oxidation in water treatment is a chemical process that converts harmful contaminants into less toxic, stable, or inert substances	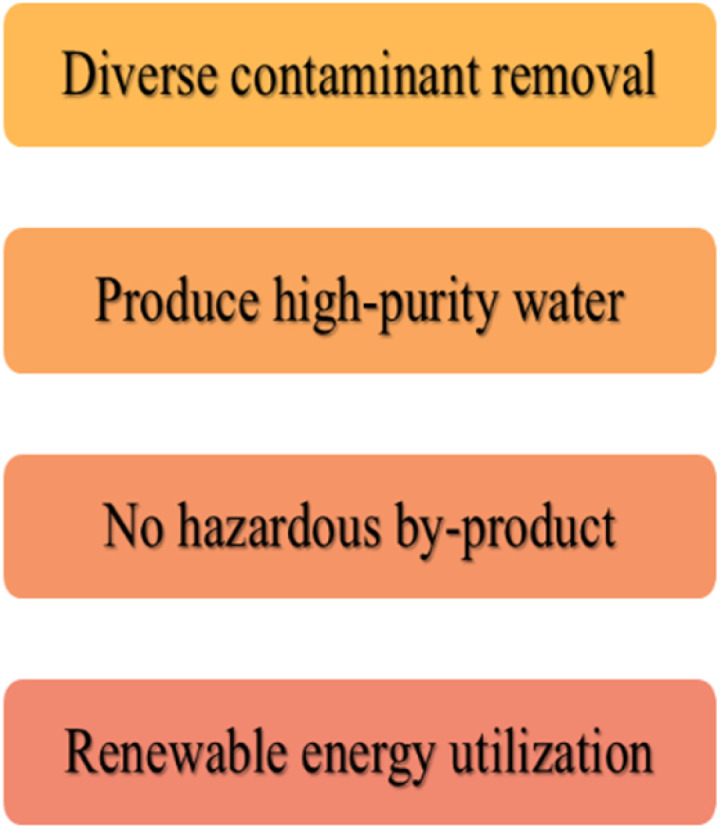	High operational cost and energy requirements, difficult monitoring, production of toxic intermediates, radical scavenging by nontarget compounds, trouble in heavy metal removal, operational errors, absence of standards, and limitations in scaling up, hindering its practical implementation	50
Precipitation: precipitation is a chemical process in which dissolved metal ions are transformed into insoluble compounds through pH adjustment, forming solid particles that can be separated from the solution	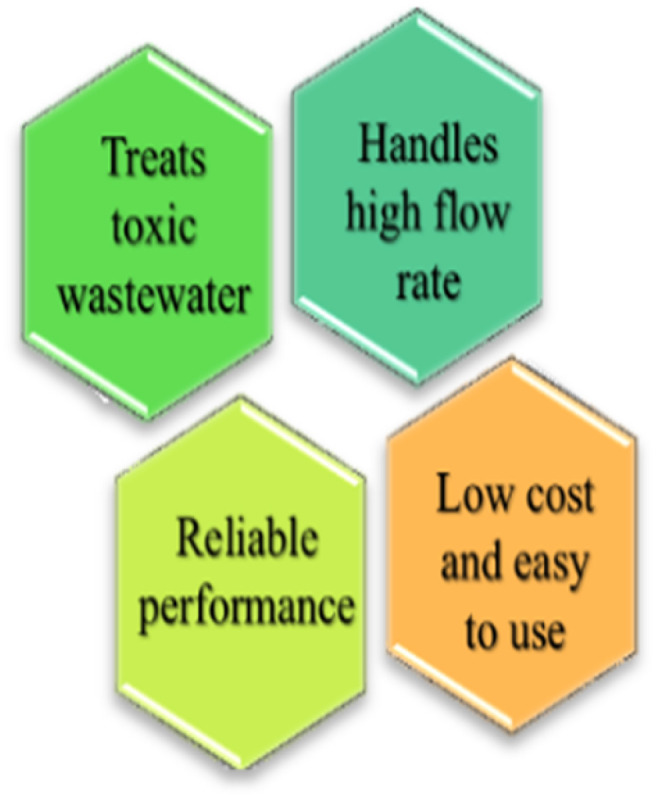	Produces a large amount of sludge, which requires proper disposal, depends on chemical inputs, which additionally increase the cost, and its efficiency is highly dependent on accurate dosing and mixing	44 and 47
Membrane processes: these are physical separation methods that purify water by selectively removing contaminants without any chemical addition or phase change	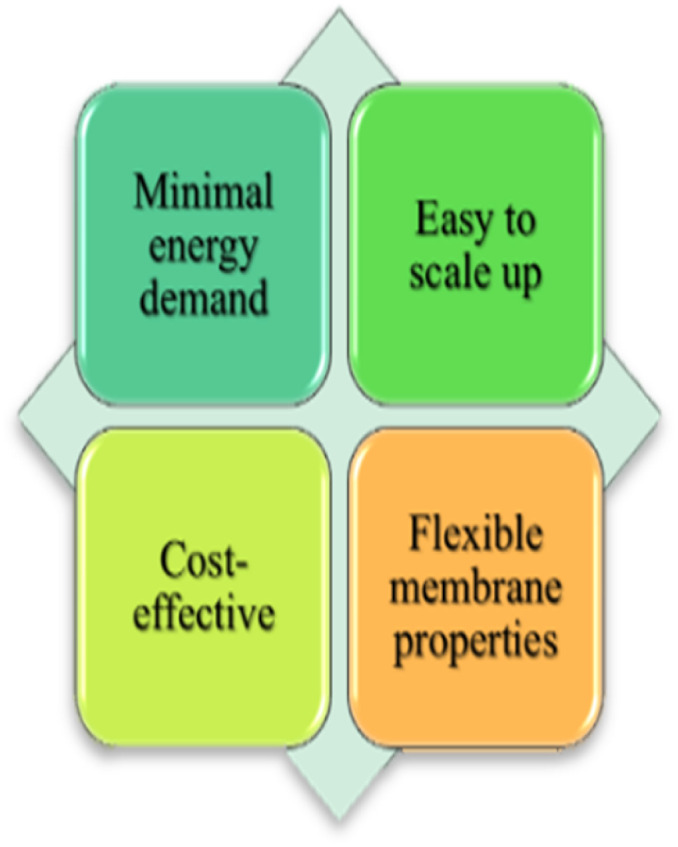	Critical challenges associated with membrane processes include high energy consumption, intensive use of chemicals, fouling, costly cleaning, and residual toxicity	51–53
Adsorption: adsorption in water treatment is a surface phenomenon through which pollutants stick to a solid adsorbent either by physisorption or chemisorption	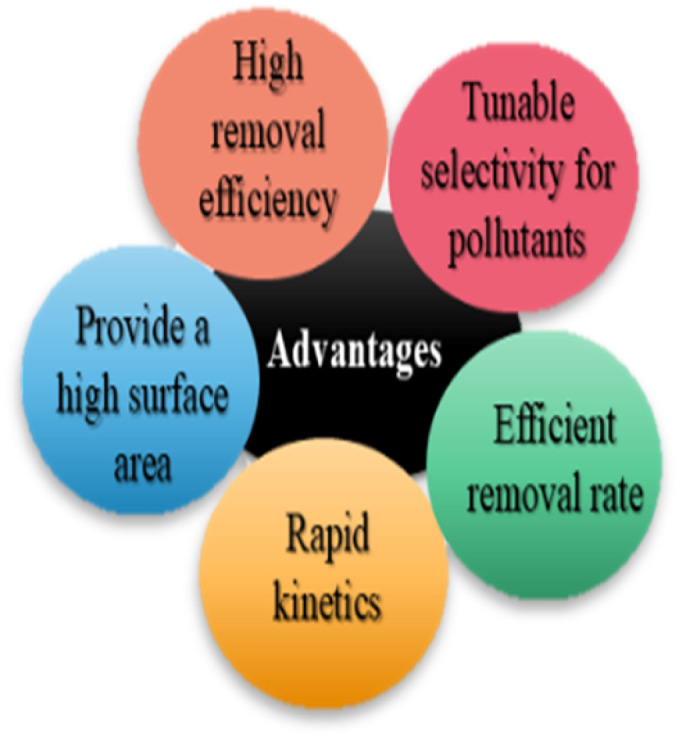	Faces drawbacks such as high production costs, reduced efficiency under varying acidic or basic conditions, potential leaching of pollutants that may cause secondary pollution, sensitivity towards water chemistry and organic matter, and stability issues in real-world applications	54

Adsorption is generally chosen for the removal of organic pollutants because of its ease of operation and versatility, although there are several challenges that make its large-scale use challenging. Adsorption is a mass transfer process in which molecules accumulate on the surface of solids instead of diffusing into the bulk phase. Its two main modes of action are chemisorption, which happens through stronger, usually irreversible chemical interactions, and physisorption, which happens through weak, reversible van der Waals forces.^55^ Additionally, the adsorption removal efficiency is significantly influenced by the physicochemical properties of the target contaminant. However, adsorbent surface chemistry, porosity, functional groups, adsorbate size, polarity, and concentration present challenges for the adsorption of organic pollutants. Furthermore, the adsorption efficiency is further complicated by variables such as pH, temperature, and ionic strength, which continuously make it challenging to achieve selectivity, scalability, and economic feasibility in real-water pollutant removal applications.^56^ The competitive adsorption effect, which arises when many adsorbate species compete for the few available adsorbent surface sites, is another difficulty in the adsorption process. Lower selectivity results from increasing competition amongst adsorbates when their initial concentration in water is increased.^57^ These limitations become particularly important in the removal of synthetic colors, agrochemicals, pharmaceuticals, and personal care items, because they have complex and inert molecular structures and different physicochemical behaviors in water environments.

Despite its drawbacks, adsorption is still the most popular method for water remediation because of its great capacity and simple operation. In this work, we specifically address the use of algae to remove organic pollutants from water. The primary mechanisms of algal biosorption are complexation and electrostatic attractions, with the cell wall possessing functional groups such as phosphate, hydroxyl, carboxyl, and amino groups that provide potent interactions that enable the binding of pollutants and the intake of nutrients,^1^ which we will discuss in a later section.

## 3. Algae as a sustainable solution for environmental remediation

### 3.1 Algae

Algae are photosynthetic thallophytes that are found in a variety of habitats. They are vital to the ecosystem balance and sustainable biotechnological advancements because they function as primary producers and oxygen generators with applications in food, bioresources, and environmental detoxification.^58^ Algae are the base of life in the ecosystem as they perform primary production, support trophic networks, and maintain biodiversity. Their cellular composition consists of essential biomolecules in the form of proteins, lipids, cellulose, carbohydrates, minerals, and vitamins, besides unique bioactive compounds such as pigments and polysaccharides, which make algae both ecologically and biotechnologically important.^59^ They differ drastically in their morphology as well as physiology and can be as small as single-cell organisms such as *Chlorella* and *Spirulina* or as huge multicellular ones such as *Sargassum*, *Ulva*, and *Laminaria*.^60,61^ The algae found in fresh water, seas, and on land can withstand changes in light, nutrient, and salt concentration. From a taxonomical point of view, the following five groups are recognized for algae: Cyanophyta (blue-green algae or cyanobacteria), Chlorophyta (green algae), Phaeophyceae (brown algae), Rhodophyta (red algae), and several other classes such as Bacillariophyceae (diatoms) and Dinophyceae (dinoflagellates).^62^ These separations are mainly determined by the differences in their pigments, storage products, cell wall components, and ultrastructural features.

Algal cells, which are the main contributors to more than half of the photosynthesis globally, not only maintain the food chain and recycle CO_2_ and nutrients through light-driven metabolism, but also act as eco-functional agents in phycoremediation, hence facilitating pollutant removal and biomass recovery from wastewater, along with contributions to sustainable energy, food, and carbon mitigation strategies.^63^

### 3.2 Functional benefits of algal biomass precursors

Microalgae are photosynthetic microorganisms that quickly convert sunlight and CO_2_ into proteins, lipids, and carbohydrate-enriched biomass; their rapid growth and carbon fixation capabilities make them a feasible solution for carbon cycling, CO_2_ mitigation, and oxygen production problems of a global nature, and therefore they are the major contributors to environmental sustainability.^64–66^ Microalgae propagate mutual benefits by synthesizing bioactive compounds with the potential of therapeutics, furnishing nutrient-rich biomass, facilitating cosmetic formulations, and energizing environmental remediation through wastewater treatment and fertilizer applications, all of which are adopted from their efficient photosynthetic metabolism.^67^ Biochar derived from algae produced by low-temperature pyrolysis in an oxygen-deficient environment has a number of beneficial properties, including increased surface area, mineral richness, and carbon stability. Additionally, its adaptability to various feedstocks and its inexpensive, low-cost, eco-friendly production highlight its potential for sustainable applications in resource recovery, advanced material development, and environmental detoxification.^68,69^

### 3.3 Methods for the modification of algae

**Fig. 2:**
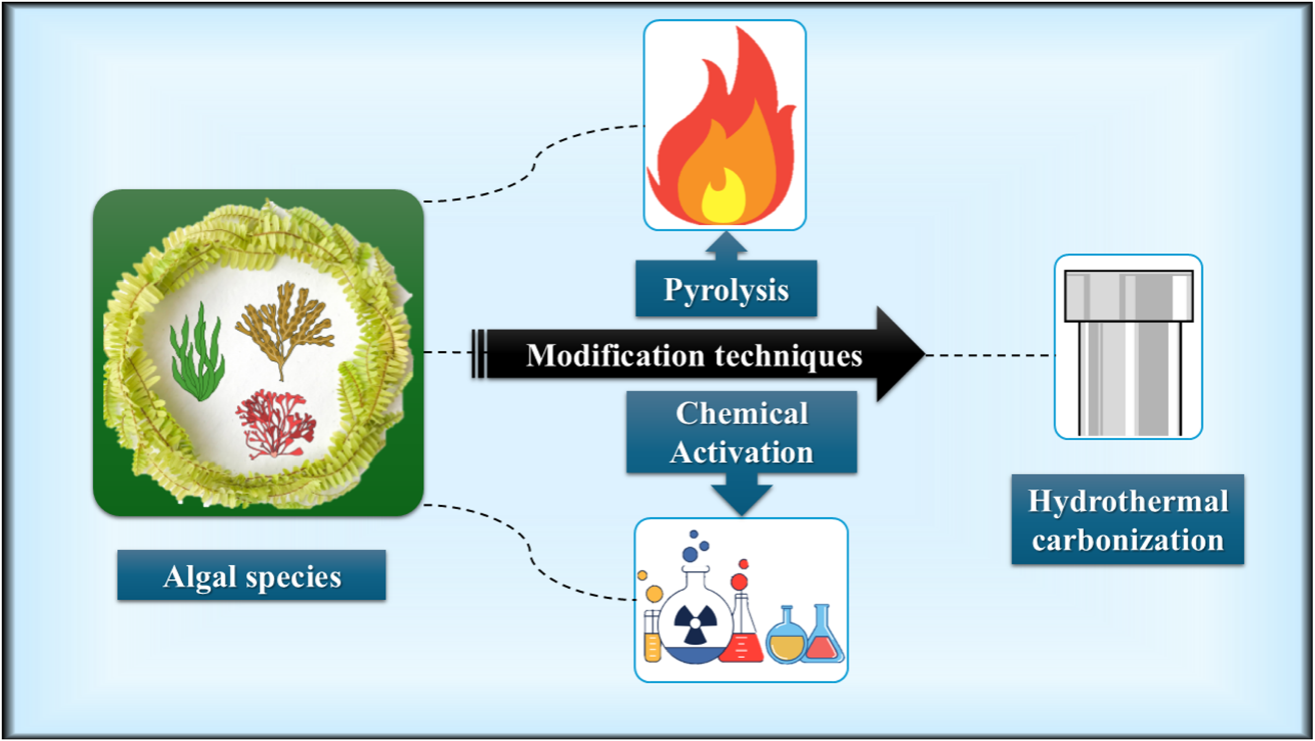
Schematic of the algal modification pathways.

This section outlines the strategic alteration techniques utilized to accelerate the conversion of algal species into carbon-rich materials, as illustrated in Fig. 2. The main objective of these interventions is to improve their physicochemical properties, which will increase their efficacy as pollutant adsorbents and improve their overall performance in water purification procedures.

#### 3.3.1 Pyrolysis

A thermal degradation technique called pyrolysis uses heat to chemically break down materials. It is a process that breaks down organic materials such as polymers, biomass, and waste to produce biochar, condensable oils, and gases. It is often done in the absence of oxygen or with very little oxygen.^70^ In addition to producing value-added materials, this technique advances environmental sustainability by creating carbon-rich products that may be used to purify water, hence reducing the levels of contaminants in aquatic settings.

#### 3.3.2 Chemical activation

Chemical activation includes the process of impregnating biochar with a chosen chemical agent (such as alkalis: KOH, NaOH, and K_2_CO_3_; acids: H_2_SO_4_, and H_3_PO_4_; and alkaline earth metal salts: particularly ZnCl_2_ and KOH) under an inert nitrogen environment, to induce structural changes and increase its porosity by controlled thermal decomposition.^71^ Chemical activation is mainly favoured because of its fast and efficient mode of operation; it can result in a shorter processing time, higher product yield, lower heating requirements, and better pore structure development properties, which collectively improve the functional performance of the resulting carbonaceous materials.^72^

#### 3.3.3 Hydrothermal carbonization

Hydrothermal carbonization (HTC) is a thermochemical conversion technique in which the material is placed in a sealed water medium at high temperature (usually the temperature is in the range of 200–300 °C) and self-generated pressure.^73^ When algae biomass is subjected to HTC, it goes through several stages of the same processes, namely hydrolysis, dehydration, decarboxylation, deamination, aromatization, and polymerization, collectively contributing to the production of hydrochar having an increased carbon content and structure stability.^74^ HTC is capable of turning biomass with a high moisture content into carbon-rich solids under relatively low thermal and pressure conditions. Hence, energy can be saved, the process is easy, and almost any type of feedstock can be used.^75^

## 4. Adsorption mechanisms

Algal biomass contains divalent cations such as Ca^2+^ and several polar functional groups, such as amide, ketonic, hydroxyl, and phenolic moieties, all of which supply numerous active sites for pollutant adsorption and significantly contribute to the environmental remediation process.^76^ The adsorption mechanism of carbon-rich materials is basically regulated by their elemental composition, pore structure distribution, and molecular framework, while pollutant uptake is mainly driven by surface-mediated interactions between the adsorbent and the physicochemical features of the carbonaceous matrix.^77,78^ In general, the adsorption of organic contaminants is a multistep process that includes surface interactions, progressive multilayer accumulation, and eventual pore diffusion, which is controlled by mechanisms such as electrostatic interactions, hydrogen bonding, π–π interactions, and complex formation.^6,79,80^ Conversely, divalent cations can drastically alter pollutant adsorption if they participate in ion exchange and form cation bridges that enhance molecular binding. Even though there is minimal direct proof in algal-derived adsorbents, these processes probably occur alongside pollutant stabilization *via* surface-mediated interactions.

**Fig. 3:**
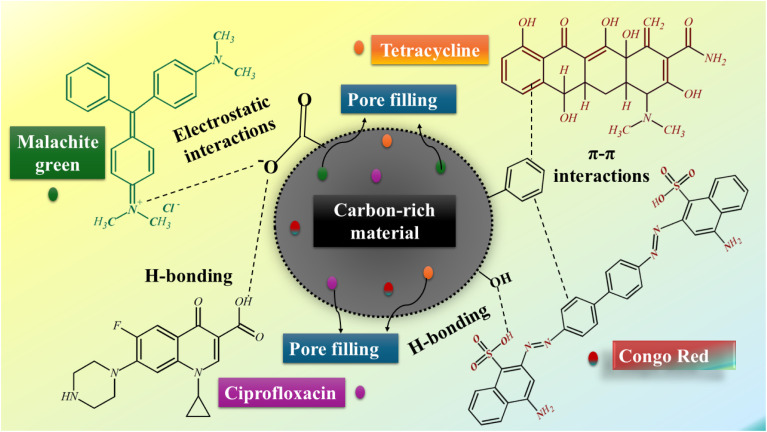
Pictorial representation of the proposed mechanism of pollutant removal.

The reason algal-based adsorbents can efficiently adsorb organic pollutants through multiple mechanisms is primarily derived from the chemical composition of their feedstock, combined with the synergistic effects of chemical heterogeneity and porous structure imparted by the thermochemical conversion process. Firstly, algae are rich in proteins, polysaccharides, and lipids, and their inherent polar functional groups, such as hydroxyl (–OH), carboxyl (–COOH), and amine (–NH_2_), provide natural sites for the adsorption of organic pollutants through electrostatic attraction and hydrogen bonding.^4,68,81^ During hydrothermal carbonization, pyrolysis, and chemical activation, these groups are converted into active sites for ligand binding or hydrogen bonding. Meanwhile, biomass forms sp^2^ hybridized carbons through deoxy-decarboxylation and arylation, which provide a structural foundation for π–π interactions with pollutants containing benzene rings.^75,82^ Secondly, natural minerals (*e.g.*, Ca^2+^, Mg^2+^, and K^+^) in algae are partially retained after carbonization or are present on the surface of the adsorbent in the form of carbonates and oxides.^69,70^ They not only allow the adsorption of cationic pollutants by ion exchange, but may also coordinate with the electron-rich groups of organic pollutants, thus enhancing the complexation and immobilization of polar organics. Moreover, defects and a large number of oxygen- or nitrogen-containing functional groups may be further introduced into the carbon skeleton through enhancement strategies such as chemical activation (*e.g.*, KOH and H_3_PO_4_) or heteroatom doping (*e.g.*, N and S). These strategies not only significantly increase the surface charge density, reinforcing electrostatic interactions, but also act as electron donors or acceptors, adjusting the π-electron cloud density and thus enhancing the π–π interaction.^71^ Additionally, the acidity and point of zero charge (pHpzc) of algal-based adsorbent surfaces can be modulated during preparation so that they exhibit variable charges under different pH conditions, thereby selectively adsorbing organic pollutants with surface polarity by electrostatic attraction.^68^ Finally, the microporous and mesoporous structures of algal-based adsorbents provide both a high specific surface area to improve the adsorption performance and ensure less diffusion resistance during the solution mass transfer process, so that chemical interactions and physical adsorption synergize to complete the multistep adsorption process from the surface to diffusion within their pores.^6,7^ Therefore, algal-based adsorbents are essentially multifunctional materials integrating polar sites, aromatic structures, inorganic mineral components, and abundant porosity, and their multiple adsorption mechanisms do not exist in isolation but work synergistically in the organic pollutant removal process. In summary, these adsorption mechanisms are vividly expressed in Fig. 3.

Based on the in-depth understanding of the above-mentioned adsorption mechanisms, the advantages of adsorption can be amplified, and the selectivity and practicability can be improved through targeted material design strategies. Also, the synergistic removal mechanism can be strengthened by regulating the preparation parameters according to the molecular characteristics of the target pollutants. Firstly, nitrogen- and oxygen-rich algal strains or complexes with multifunctional materials are preferred in the selection of precursors to naturally provide more sites where hydrogen bonding and complexation can be formed. Secondly, a balance between promoting aromatization to enhance the π-electron cloud and preserving the pore structure can be achieved by carefully controlling the thermal treatment temperature, time, and heating rate, thereby combining strong interactions with pollutants with a high specific surface area.^70^ For example, to efficiently remove aromatic contaminants (*e.g.*, tetracycline), pyrolysis at higher temperatures (>700 °C) could be used to enhance the graphitization of the carbon material and thus maximize the π–π interactions.^70^ Thirdly, pHpzc and polar sites may be adjusted through activation or surface engineering to achieve the selective adsorption of specific ionic or polar pollutants. For complexation-dependent targets, native minerals can be retained or gently doped with non-toxic metals (Fe, Mn, Cu, *etc.*) to form stabilizing coordination sites. For example, for ionic or highly polar pollutants (*e.g.*, dyes or pesticides), surface charge can be modulated and specific metal sites introduced by acid treatment or metal salt impregnation to optimize electrostatic interactions and surface complexation. Fourthly, combined with the advantages of KOH activation, the templating method or acid etching technique could build mesoporous and microporous complementary porous structures to optimize the adsorption energy and mass transfer rate.^71^ Meanwhile, this not only introduces abundant surface functional groups but also enhances hydrogen bonding and electrostatic adsorption. Finally, environmental compatibility and renewability must be considered at the same time when designing materials. Additionally, for mechanism-driven experimental optimization that can be effectively translated into scalable and sustainable engineering solutions, multi-cycle and interference ion assessments in complex water bodies should be conducted. For example, the additional adsorption sites provided by compositing magnetic nanoparticles (*e.g.*, Fe_3_O_4_) with alginate-based materials also confer magnetic separation properties, which facilitate recycling and reuse after adsorption.^69^

## 5. Applications of modified algal-based adsorbents

Here, the focus is on how algal-based adsorbents have been utilized to detoxify various organic contaminants such as synthetic dyes, pharmaceutical residues, phenolic compounds, and agrochemical pollutants. Algae-derived materials, with surface functionality and porosity, have been used as a source of adsorbents, making their use a desirable green and very effective technique for the removal of challenging organic pollutants in water bodies.

### 5.1 Removal of pharmaceutical pollutants

**Fig. 4:**
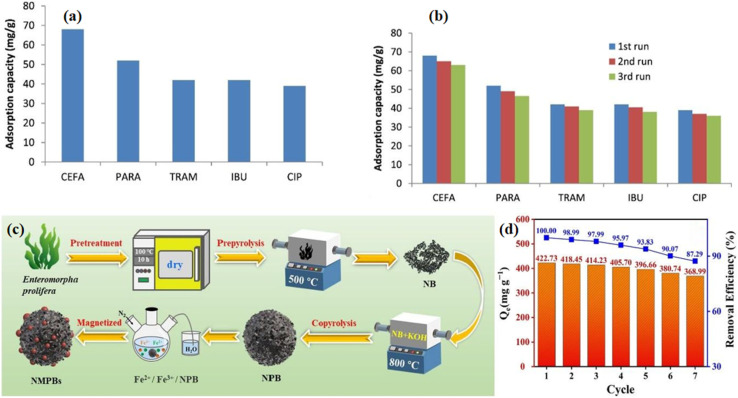
(a) Efficient adsorption of drugs by modified biomass and (b) reusability of MAB in pharmaceutical wastewater treatments. (c) Prepyrolysis, copyrolysis, and coprecipitation synthesis route and (d) reuse efficiency of NMPBs. Reproduced from ref. 84 and 89, with permission from ELSEVIER,^84,89^ copyright 2018 and 2024.

Algae-derived adsorbents are very effective for the removal of pharmaceutical pollutants. For example, magnetic biochar prepared from microalgae by hydrothermal carbonization with iron loading exhibited an improved tetracycline adsorption capacity of up to 95.86 mg g^−1^ at room temperature under optimized parameters (pH 6.8 and adsorbent mass of 0.20 g/100 mL).^83^ Pseudo-second-order kinetics and the Langmuir isotherm showed the best fit for the adsorption data. The main reasons for the higher efficiency were hydrogen bonding, numerous oxygen-containing groups, iron-induced bridging effects, and an increased surface area of 122.8 m^2^ g^−1^. Furthermore, owing to its remarkable reusability, it maintained over 96% recovery after three consecutive cycles, proving its capacity to stabilize pollutants in natural solutions. In a different experiment, the biomass of *Scenedesmus obliquus* was chemically modified by an alkaline treatment for the adsorption of pharmaceutical pollutants from aqueous environments.^84^ This modified algal biomass (MAB) exhibited notable efficiency in adsorbing various pharmaceutical contaminants from aqueous media, including tramadol (TRAM), paracetamol (PARA), cefadroxil (CEFA), ciprofloxacin (CIP), and ibuprofen (IBU). The adsorption capacities were measured to be as follows: 42 mg g^−1^ for TRAM, 58 mg g^−1^ for PARA, 68 mg g^−1^ for CEFA, 39 mg g^−1^ for CIP, and 42 mg g^−1^ for IBU, as shown in Fig. 4(a).

More specifically TRAM adsorption was observed at neutral pH, following pseudo-order-kinetics. The Langmuir isotherm fitting confirmed monolayer coverage, yielding a maximum adsorption capacity of 140.25 mg g^−1^. The adsorption of pharmaceuticals on the modified algal biomass is achieved through hydrophilic interactions between the functional groups of biosorbent surface and the MAB, which exhibited very high reusability, as it retained high adsorption efficiency with only a slight decrease of 4.5% after three consecutive cycles, as shown in Fig. 4(b), confirming its use as a sustainable biosorbent for pharmaceutical wastewater treatment. A carbon-rich material, named CMBB@H_2_SO_4_, was synthesised from *Bifurcaria bifurcata* (BB) algae *via* an ultrasonic-assisted pyrolysis process, and then chemically activated with sulphuric acid to improve its surface functionality and adsorption capacity.^85^ This material was tailored explicitly for the efficient removal of aspirin from aqueous solutions. The adsorption of aspirin on the prepared material is spontaneous, occurs naturally without the need for external energy, is exothermic, and is controlled by physical interactions. The adsorption kinetics of aspirin on CMBB@H_2_SO_4_ followed pseudo-first-order kinetics, indicating physisorption, while its equilibrium data fitted the Langmuir isotherm, thus confirming monolayer adsorption on a homogeneous surface. This material exhibited an impressive performance, which remained almost unchanged for 10 reuse cycles, with only a minimal 4% efficiency loss. This synthesized material exhibited a high surface area of 898.2 m^2^ g^−1^ and achieved an excellent adsorption efficiency up to 2633.04 mg g^−1^ under optimized conditions (adsorbent mass of 0.02 g/100 mL, pH 3.4, and temperature (temp.) of 298 K), and at the same time, it can be used as a cost-effective alternative to remove pharmaceutical pollutants from aqueous solutions. To further optimize the adsorption process, response surface methodology (RSM) in combination with central composite design (CCD) was used to evaluate the combined effects of the operational parameters. The quadratic model was statistically significant and had a very high coefficient of determination, indicating a very good match between the predicted and the experimental results. The interaction analysis showed that a simultaneous increase in adsorbent dose and pH would result in the most substantial improvement in aspirin removal efficiency. The optimum conditions correspond to pH 3.4, adsorbent dosage of 20 mg, and aspirin concentration of 150 mg L^−1^. The significant influence of pH is consistent with the fact that surface charge and protonation and deprotonation mechanisms play a major role in aspirin adsorption.

In general, RSM provides a reliable tool that not only fits the studies of mechanism but also facilitates the scaling-up of the process. Another study used an adsorption technique to assess the removal of pharmaceutical pollutants, specifically aspirin and ketoprofen, from water using porous carbon produced from *Laminaria digitata* that was chemically activated with NaOH (PCLD@NaOH).^86^ Under ideal conditions (adsorbent dosage: 0.02 g/100 mL, pH: 3.4, temp.: 25 °C), the modified material exhibited a significant adsorption capacity for aspirin (970.88 mg g^−1^) compared to ketoprofen (443.45 mg g^−1^). It also showed a specific surface area of 799 m^2^ g^−1^. The adsorption of aspirin and ketoprofen on the prepared material followed Avrami-fractional kinetics and the Liu isotherm. This material showed very high reusability and retained more than 90% of its adsorption capacity for aspirin and ketoprofen through 5 regeneration cycles, with just a minor decline in performance loss; therefore, its structural stability and long-term adsorption capacity were confirmed. The study also integrates density functional theory (DFT) and RSM to rationalize and optimize the adsorption process, in addition to demonstrating high adsorption capacity and reusability. The lower HOMO–LUMO gap (HOMO: highest occupied molecular orbital and LUMO: lowest unoccupied molecular orbital), higher electrophilicity index, greater softness, and higher chemical potential of aspirin all favour electron transfer and its adsorption on the substrate, and showed that it is more reactive than ketoprofen, according to the DFT analysis. Complementary optimization by RSM confirmed these results, as statistically valid quadratic models showed a very good agreement between the predicted and experimental data. Temperature was the most influential parameter since higher temperatures lowered the adsorption efficiency, while the adsorbent dose and pH also had some influence on the process. Under the optimum conditions, the removal efficiencies between the two drugs were 86.12% for ketoprofen and 95.33% for aspirin, thus correlating the higher reactivity of aspirin with its better adsorption performance and demonstrating RSM to be a reliable predictive tool for process enhancement.

Choi *et al.* (2020) used the pyrolysis technique at various temperatures to formulate biochars from the microalgae species *Spirulina* sp. to remove tetracycline (TC) from water.^87^ The aromaticity, surface area, and hydrophobicity of the biochar were significantly improved by an increase in the pyrolysis temperature, with SPAL-BC750 exhibiting the best properties. This material showed intense crystallinity and a variety of functional groups (C–N, C–O, CH_2_, and CO_3_^2−^), resulting in a very high TC adsorption capacity of 132.8 mg g^−1^ under the optimised conditions (adsorbent mass of 0.005 g mL^−1^, pH 6, and temp of 20 °C). The adsorption data were best represented by the Langmuir isotherm and pseudo-first-order kinetics. The adsorption process was dominated by hydrophobic interactions, π–π stacking, electrostatic attractions, and metal complexation, indicating that the material could be a low-cost adsorbent for wastewater contaminated with antibiotics. In addition, statistical evaluation using Pearson's correlation analysis showed that the pyrolysis temperature had a significant negative impact on most mineral contents (Ca, Mg, N, P, Fe, and K) but a positive impact on ash content, surface area, H/C ratio, and TC adsorption capacity. The increase in adsorption capacity at higher pyrolysis temperatures was attributed to the increase in carbon content, decrease in H and O, more ash, and a large surface area. In particular, the H/C ratio showed a strong negative correlation with temperature; thus, the achievement of hydrophobicity and aromaticity was more efficient at higher pyrolysis levels. The adsorption capacity was majorly influenced by surface area and H/C ratio, which is consistent with the increase in TC removal through higher pyrolysis temperatures, large surface areas, and lower H/C ratios. In another study, zeolite-modified biochar derived from *Sargassum crassifolium via* pyrolysis and slurry coating showed enhanced ciprofloxacin removal due to the increase in active sites and enhancement in surface area up to 124.359 m^2^ g^−1^.^88^

This hybrid material achieved a ciprofloxacin adsorption capacity of 93.65 mg g^−1^, mainly through electrostatic interactions and hydrogen bonding, with the pH range of 6.5–8 being the region of maximum efficiency where the processes were one of chemisorption and physisorption. The adsorption data showed that the Freundlich model fitted best, while the kinetics were well described by the pseudo-second-order and Elovich models. This modified biochar presents a porous and inexpensive way of filtering domestic greywater in soaking pits; thus, it can get rid of personal care products and pharmaceutical residues. Also, since it can be produced very quickly at home, it is an ideal solution for coastal communities. Wu *et al.* (2021) synthesized biochar from *Enteromorpha prolifera* and modified it with potassium hydroxide to enhance its surface properties.^90^ After modification, this material showed a large surface area of 2172.08 m^2^ g^−1^. The final material showed the capability of efficiently removing sulfamethoxazole, a typical antibiotic contaminant. After modification of the synthesized material, it demonstrated a very high sorption capacity for sulfamethoxazole, reaching as high as 744 mg g^−1^, which was mainly controlled by both physical and chemical interactions such as pore filling, hydrogen bonding, electrostatic forces, and π–π interactions. The pseudo-second-order kinetics for the sorption of sulfamethoxazole onto the modified material indicated that surface interactions were mostly unimportant in the adsorption process. Both the Freundlich (multilayer adsorption) and Langmuir (monolayer adsorption) models, which suggest the dual adsorption behaviour of the system, are well-aligned with the isotherm fitting. The biosorbent with optimized properties maintained 94% adsorption efficiency after five reuse cycles, demonstrating powerful regeneration ability and a stable performance for the eco-friendly removal of antibiotic pollutants from wastewater.

**Fig. 5:**
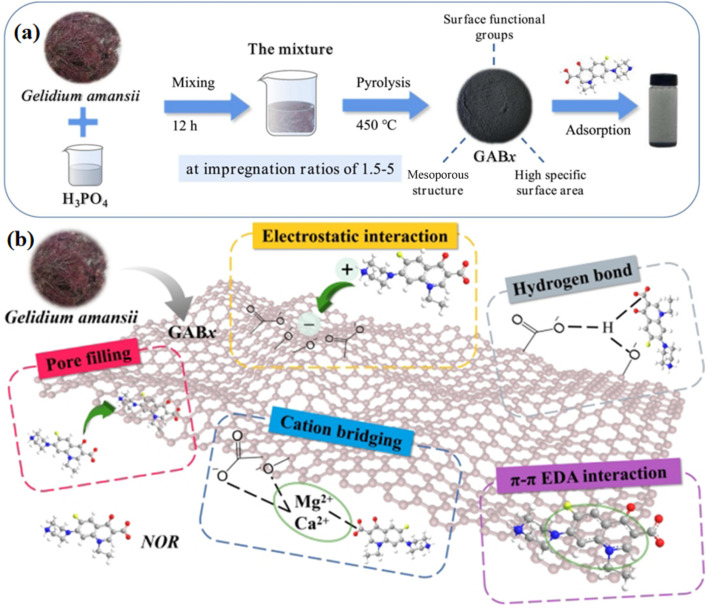
Schematic of (a) GAB synthesis and (b) NOR adsorption mechanism, reproduced from ref. 92, with permission from ELSEVIER,^92^ copyright 2022.

Another study has also been done on biochar production from *Ascophyllum nodosum* through the hydrothermal carbonization technique and ZnCl_2_ activation, enabling ciprofloxacin adsorption (150–400 mg g^−1^) over a wide range of conditions (adsorbent dosage of 50 mg L^−1^, pH range of 3–11, and temperature range of 5–45 °C).^91^ The efficiency was controlled by the ionic composition, pH, and water matrix, which essentially highlights algal biochar as a green and very effective adsorbent for the removal of antibiotics from water. Moreover, pseudo-second-order kinetics and the Langmuir isotherm best fitted the adsorption data. Another study explored mesoporous biochars, which were developed by H_3_PO_4_ activation from *Gelidium amansii* (GAB) for the removal of norfloxacin (NOR) from seawater, as shown in Fig. 5(a).^92^ GAB3, the product obtained at an acid-to-biomass ratio of 3, exhibited a specific surface area of 641.33 m^2^ g^−1^ and a maximum adsorption capacity for norfloxacin of 166.48 mg g^−1^ in seawater and 201.80 in deionised water at a temperature of 450 °C with an adsorbent dosage of 0.5 g L^−1^, which is attributed to its increased mesoporosity and decreased number of oxygenated groups. Moreover, it was able to retain ∼80% of its NOR removal efficiency after four reuse cycles, indicating its strong reusability and structure stability during repeated use. While adsorption was slightly inhibited by increased salinity, probably because of ionic interference, the presence of humic acid had almost no effect, which means that GAB3 remains stable under natural marine conditions. Adsorption was enabled by a set of processes that involved electrostatic interactions, cation bridging, pore filling, π–π interactions, and hydrogen bonding, which were enhanced by surface charge, as shown in Fig. 5(b). These findings support the potential use of algal-biochars as green and large-scale materials for the removal of antibiotics in marine ecosystems.

Tetracycline is one of the most common antibiotics found in water ecosystems, which has been removed using activated carbon produced from *Ulva prolifera via* low-temperature carbonization.^93^ The developed adsorbent showed a rough, porous surface and a large surface area (197.53 m^2^ g^−1^), which enhanced its ability to capture tetracycline molecules from water with an adsorption capacity of 54.04 mg g^−1^ in a batch experiment under optimized conditions (adsorbent mass of 0.5 g L^−1^, pH 6, temperature of 30 °C). The isotherm data were well represented by the Langmuir and Freundlich models, while the kinetic data fitted well with the pseudo-second-order model. The adsorption was mainly a result of chemical interactions, which were supported by the surface composition of the material, showing the effectiveness of this low-cost algal-based adsorbent for antibiotic remediation. Hourcade *et al.* (2022) demonstrated that nitrogen-doping through melamine improved the structural and functional properties of microalgae-derived biochars drastically, which were obtained by one-step H_3_PO_4_-assisted pyrolysis of a wild microalgal mixture containing *Coelastrum* ssp., *Desmodesmus* ssp., *Scenedesmus* ssp., and *Chlorella* ssp.^94^ The doping treatment increased the surface area from 324 to 433 m^2^ g^−1^, microporosity, and graphitization, and nitrogen and oxygen functionalities were enriched. These changes improved the adsorption capacity for acetaminophen; the doped biochar achieved 120.7 mg g^−1^ compared to 101.4 mg g^−1^ for the non-doped biochar under optimized conditions (adsorbent mass of 2 g L^−1^, pH 6, and temperature of 23 °C). Filling of pores was the main adsorption mechanism. In addition, the biochars were capable of removing up to 74% of pollutants from synthetic effluents, underscoring their potential in the field of sustainable water treatment applications. Qin *et al.* (2023) prepared porous carbon-biochar composites (PC/PB) from *Enteromorpha prolifera* through pyrolysis and potassium/iron citrate-assisted activation at 800 °C, exhibiting a hierarchical pore structure with high surface area and pore volume.^95^ Among them, the HCl-washed A-PC/PB composite showed a high specific surface area of 1414.89 m^2^ g^−1^ and excellent adsorption performance for sulfamethoxazole, achieving a maximum capacity of 844 mg g^−1^. The adsorption was primarily governed by pore filling, supported by electrostatic attractions, hydrogen bonding, and π–π interactions. The sorption of the pollutant was well described by both the Freundlich and Langmuir isotherm models, while the adsorption kinetics followed the pseudo-second-order model. The reusability test confirmed the stability of the absorbent, maintaining 95.05% removal efficiency and 109.35 mg g^−1^ adsorption capacity even after four cycles, underscoring its potential for sustainable water remediation.

In the study by Xu *et al.* (2023), the composite biochar EC_A_-B was prepared from *Enteromorpha* and *Chlorella vulgaris* through pyrolysis and NaOH activation of the sample. It showed a very good tetracycline adsorption capacity (376.878 mg g^−1^) within the pH range of 3–9 at 50 °C, which was mainly attributed to intraparticle diffusion.^96^ The prepared material exhibited a specific surface area of 583.329 m^2^ g^−1^, and the adsorption data fitted best with pseudo-second-order kinetics and the Langmuir isotherm. After five cycles, the adsorbent still retained over 80% of its removal efficiency, which is proof of its high reusability, making it a great solution for wastewater treatment. Chen *et al.* (2024) synthesized nitrogen-doped magnetic porous biochars (NMPBs) from *Enteromorpha prolifera via* prepyrolysis, copyrolysis, and coprecipitation techniques, as shown in Fig. 4(c). These porous materials offer high surface areas up to 1531 m^2^ g^−1^, an enriched nitrogen content, and strong magnetization, exhibiting an exceptional performance for the adsorption of sulfamethoxazole through mechanisms such as hydrogen bonding, π–π interactions, pore filling, and electrostatic attractions.^89^ These NMPBs demonstrated a high affinity for sulfamethoxazole, achieving a maximum adsorption capacity of 502 mg g^−1^ and also exhibited strong magnetic separability and notable reusability, maintaining an 87% removal efficiency even after seven adsorption and desorption cycles, as shown in Fig. 4(d). Furthermore, the adsorption data was well described by the Freundlich isotherm, and the kinetics fitted both pseudo-first- and second-order models. This work highlights the potential of converting marine biowaste into functional biochars, offering sustainable routes for advanced environmental remediation applications. Sun *et al.* (2025) prepared a zeolite-like algal biochar using *Sargassum horneri* as the precursor and doping heteroatoms such as nitrogen, sulphur, oxygen, silicon, and aluminium to enhance its surface functionality and structural properties.^97^ The resulting material exhibited high porosity, a large number of oxygen-containing functional groups and a high surface area (1137.60 m^2^ g^−1^), enabling the efficient adsorption of CIP and TC with the maximum capacities of 352.936 and 265.385 mg g^−1^, respectively (at the adsorbent dosage of 0.02–0.10 g L^−1^, pH range of 3–11, and temperature of 20–60 °C). The adsorption behaviour was best fitted by the Freundlich isotherm and pseudo-first-order kinetics.

**Fig. 6:**
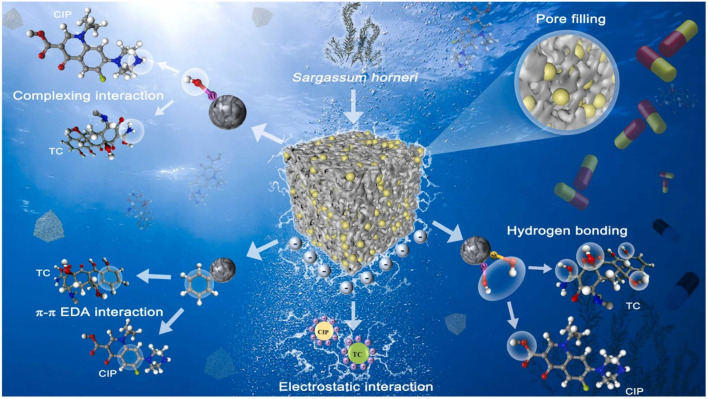
Pictorial representation of the adsorption mechanism of CIP and TC, reproduced from ref. 97, with permission from ELSEVIER,^97^ copyright 2025.

The adsorption occurred through various mechanisms such as hydrogen bonding, pore filling, electrostatic attraction, complexation, and π–π electron-donor–acceptor interactions, as shown in Fig. 6. These findings emphasized the strong potential of the prepared material for the removal of antibiotics from antibiotic-contaminated water. Furthermore, DFT simulation analysis was performed to clarify the adsorption mechanism of the prepared adsorbents toward CIP and TC. Electrostatic potential and frontier orbital analysis were used to find the reactive sites, while five substrates were optimized. The adsorption energies confirmed that the mechanism was chemisorption, and the prepared material showed the highest affinity for CIP (−2.19 eV) and TC (−3.31 eV). The charge density difference analysis revealed enhanced electron transfer from the synthesized material to pollutants, and the electron localization function explained the hydroxyl-driven interactions with CIP and hydrogen bonding with TC. Therefore, the synergistic changes in the adsorbent (N doping, Al substitution, and hydroxylation) significantly improved its adsorption capacity owing to charge transfer and hydrogen bonding, which is consistent with the experimental data.

One more study highlighted how structural defects played a major role in improving the adsorption capacity of algal biochar for the removal of ciprofloxacin.^98^ In this study, biochar from *Ulva lactuca* was prepared *via* high-temperature carbonization and KOH activation, which resulted in the introduction of oxygen and sulphur dopants along with carbon vacancies in the material. These structural transformations increased the interaction energy and decreased the electrostatic potential, causing pollutant adsorption to be highly efficient through hydrogen bonding, pore-filling, electrostatic attractions, and π–π electron donor–acceptor interactions. Furthermore, these modifications increased the porosity and surface area (1799.23 m^2^ g^−1^), thereby leading to a very high ciprofloxacin adsorption of 695.09 mg g^−1^ under optimized conditions (adsorbent dosage of 0.004 g/100 mL, pH 7, and temperature of 25 °C). The adsorption kinetics aligned well with pseudo-second-order kinetics and the Langmuir isotherm. Notably, it is worth mentioning that the biochar maintained 69.36% of its original adsorption capability after five regeneration cycles from 91.05%; hence, its performance can be considered stable, and its reusability is promising. In addition, DFT analysis was employed to determine how oxygen and sulphur doping, along with carbon vacancies, contribute to the adsorption of ciprofloxacin on biochar. Five graphene-based models were constructed, and it was found that the individual defects decreased the local electron density, whereas the inclusion of a carbon vacancy in conjunction with oxygen and sulphur doping produced the adsorption energy with the highest absolute value (−0.38 eV). In other words, the existence of several defects provides reaction sites, enhancing the charge redistribution and lowering the electrostatic potential, consequently leading to the stronger adsorption of ciprofloxacin. Overall, this study demonstrated that defect engineering through heteroatom doping and vacancy creation significantly improves the adsorption capacity of algal-derived biochar, together with its high porosity and surface area. This research outcome provides substantial knowledge about defect engineering of marine algal biochars for efficient antibiotic removal.

A recent study on hydrochars derived from a strain of microalgae local to northern Sweden through hydrothermal carbonization at 180 °C explored their capacity to remove pharmaceuticals and personal care pollutants from water.^99^ The adsorption behaviour of the materials prepared was enhanced by alkane/alkene and aromatic structures, which were the main factors that influenced the adsorption of hydrophobic compounds such as triclosan (58.8 mg g^−1^) and bisphenol A (25.8 mg g^−1^), whereas oxygen-containing groups promoted the adsorption of cationic molecules such as trimethoprim. Conversely, negatively charged pollutants such as diclofenac and chloramphenicol had very low adsorption, which was attributed to electrostatic repulsions. These findings emphasized the ability of hydrochar derived from Swedish microalgae to be used as adsorbents for selective contaminant removal.

Algal-based material demonstrates excellent adsorption capacities for the pharmaceutical contaminants. Furthermore, their adsorption mechanism for the removal of pharmaceutical pollutants was validated by DFT/computation simulations, which provide an understanding of adsorbent interactions with pollutants at the atomic level. In addition, their adsorption efficiency and reusability were enhanced after chemical modification and metal doping. These findings suggest the possible application of algal-derived materials as a sustainable method to mitigate pharmaceutical contaminants in wastewater and open up new opportunities in environmental detoxification.

Table 2 summarizes the reported literature (2014–2025) on algae-derived adsorbents for the removal of pharmaceutical pollutants. This table briefly presents diverse algae-derived adsorbents prepared through pyrolysis, hydrothermal carbonization, and chemical activation methods. The carbonaceous material derived from *Bifurcaria bifurcata* showed the highest drug adsorption capacity for aspirin (2633.04 mg g^−1^), while activated carbon obtained from *Ulva prolifera* exhibited the lowest tetracycline adsorption capacity of 54.04 mg g^−1^. The adsorption data follows various kinetic models and isotherm models depending on the modification technique and type of pollutant. Most of the algal-derived adsorbents exhibited pseudo-second-order kinetics, indicating chemisorption as the primary mechanism. Alternatively, advanced models such as Avrami fractional and general-order models revealed that the adsorption mechanism is complex. The majority of isotherm fittings are Langmuir, suggesting monolayer adsorption; however, the Freundlich and Liu models frequently take heterogeneity into consideration. With a constant high adsorption capacity (for example, *Enteromorpha prolifera*: 744–844 mg g^−1^ and *Ulva lactuca*: 695.09 mg g^−1^) and good reusability, pseudo-second-order kinetics with the Langmuir isotherm stands out among them the most stable combination. Using algal-derived adsorbents, these studies demonstrated that chemisorption-driven monolayer adsorption was the most effective method for removing pharmaceutical pollutants.

**Table 2:** Summary of algae-derived adsorbents, highlighting their synthesis methods, pharmaceutical pollutants removal efficiencies, and regeneration performance across multiple cycles

Algal species used	Modification technique	Adsorbent derived from algal species	Specific surface area (m^2^ g^−1^)	Adsorbed pollutants	Adsorption capacities (mg g^−1^)	Optimized parameters	Adsorption kinetic models and isotherms	Reusability	Year	Ref.
Blue-green microalgae	Hydrothermal carbonization	Biochar	122.8	Tetracycline	95.86	pH: 6.8	Pseudo-second-order and Langmuir isotherm	96% after three cycles	2014	83
						Adsorbent mass: 0.20 g/100 mL				
						Room temperature				
*Scenedesmus obliquus*	Alkaline modification	MAB	—	Tramadol	140.25	pH: 7	Pseudo-second-order kinetics and Langmuir isotherm	∼95% after three runs	2018	84
						Adsorbent mass: 0.5 g/1000 mL				
						Temp.: not specified				
*Bifurcaria bifurcata*	Ultrasonic-assisted pyrolysis	Carbonaceous material	898.2	Aspirin	2633.04	pH: 3.4	Pseudo-first-order and Langmuir model	∼95% after 10 cycles	2019	85
						Adsorbent mass: 0.02 g/100 mL				
						Temp.: 293 K				
*Laminaria digitata*	Chemical activation	Porous carbon	799	Ketoprofen	443.45	pH: 3.4	Avrami fractional kinetics and Liu isotherm model	90.2%	2019	86
				Aspirin	970.88	Adsorbent mass: 0.02 g/100 mL		∼90.6% after 5 cycles		
						Temp.: 298 K				
*Spirulina* sp.	Pyrolysis	Biochar	2.63	Tetracycline	132.8	pH: 6	Pseudo-first-order kinetic model and Langmuir isotherm	60–65% over the first three cycles and declined to 37% in the fourth cycle	2020	87
						Adsorbent mass: 0.005 g/50 mL				
						Temp.: 293 K				
*Sargassum crassifolium*	Pyrolysis and slurry coating	Zeolite-modified seaweed biochar	124.359	Ciprofloxacin	93.65	pH: 7	Pseudo-second-order, Elovich, and Freundlich	—	2021	88
						Adsorbent dosage: 0.5 g/1000 mL				
						Temp.: 298 K				
*Enteromorpha prolifera*	Potassium-hydroxide modification	Biochar	2172.08	Sulfamethoxazole	744	pH range: 5–9	Pseudo-second-order kinetics and Freundlich and Langmuir isotherms	94% after five cycles	2021	90
						Adsorbent mass: 0.002 g/20 mL				
						Temp.: 298 K				
*Ascophyllum nodosum*	Hydrothermal carbonization	Biochar	49 to 1326	Ciprofloxacin	150–400	pH range: 3–11	Pseudo-second-order and Langmuir isotherm	—	2022	91
						Adsorbent dosage: 0.05 g/1000 mL				
						Temp. range: 278–318 K				
*Gelidium amansii*	Pyrolysis and H_3_PO_4_ activation	Biochar	641.33	Norfloxacin	166.48	pH: not specified	Pseudo-second-order kinetics and Langmuir isotherm	∼80% even after four reuse cycles	2022	92
						Adsorbent dosage: 0.5 g/1000 mL				
						Temp.: 723 K				
*Ulva prolifera*	Low-temperature carbonization	Activated carbon	197.53	Tetracycline	54.04	pH: 6	Pseudo-second-order kinetics	—	2022	93
						Adsorbent dosage: 0.5 g/1000 mL	Freundlich and Langmuir isotherms			
						Temp.: 303 K				
Wild microalgal consortium (*Coelastrum*, *Desmodesmus*, *Scenedesmus*, *Chlorella*)	H_3_PO_4_-assisted pyrolysis	Biochar	433	Acetaminophen	120.7	pH: 6	General-order and Liu isotherm	—	2022	94
						Adsorbent dosage: 2 g/1000 mL				
						Temp.: 296 K				
*Enteromorpha prolifera*	Citrate-mediated green activation process	Porous carbon/porous biochar (PC/PB) composites	1414.89	Sulfamethoxazole	844	pH: 6	Pseudo-second-order kinetics and isotherm fitting align with both Freundlich and Langmuir isotherms	95.05% after four cycles	2023	95
						Adsorbent dosage: 0.002 g/20 mL				
						Temp.: 298 K				
*Enteromorpha* and *Chlorella vulgaris*	Pyrolysis and NaOH activation	Biochar	583.329	Tetracycline	376.878	pH: 9	Pseudo-second-order kinetics and Langmuir isotherm	80% after five cycles	2023	96
						Adsorbent dosage: 0.1 g/200 mL				
						Temp.: 323 K				
*Enteromorpha prolifera*	Prepyrolysis, copyrolysis, and coprecipitation	Nitrogen-doped magnetic biochar	1531	Sulfamethoxazole	502	pH: 7	Pseudo-first and second-order kinetics and Freundlich isotherm	87% after seven cycles	2024	89
						Adsorbent dosage: not specified				
						Temp.: 298 K				
*Sargassum horneri*	Pyrolysis and chemical activation	Biochar	1137.60	Ciprofloxacin	352.936	pH range: 7 for CIP and 3 for TC	Pseudo-first-order kinetics and Freundlich isotherm	Cyclic regeneration over four cycles	2025	97
				Tetracycline	265.385	Adsorbent dosage: 0.02–0.10 g/1000 mL				
						Temp.: 293–333 K				
*Ulva lactuca*	High-temperature carbonization and KOH activation	Biochar	1799.23	Ciprofloxacin	695.09	pH: 7	Pseudo-second-order kinetics and Langmuir isotherm	91.05% → 69.36% by the fifth cycle	2025	98
						Adsorbent dosage: 0.004 g/1000 mL				
						Temp.: 298 K				
Microalgae	Hydrothermal carbonization	Hydrochar	15.3	Triclosan	58.8	pH: 7	Langmuir isotherm	—	2025	99
						Adsorbent mass: 0.05 g/50 mL				
						Temp.: 293 K				

### 5.2 Removal of dye pollutants

Algal-based adsorbents are very effective for the removal of dye pollutants. In a previous study, biochar was obtained *via* the thermochemical activation of residual biomass (DB) from *Spirulina platensis*, a waste biomass produced after *in situ* transesterification during biodiesel production.^100^ As a part of a closed-loop waste-to-valorisation approach, the produced biochar was tested for its capacity to remove Congo red dye from aqueous solutions. Native algae biomass (AB), commercial activated carbon (AC), and untreated DB were used in parallel batch adsorption studies, and the results showed that AC (85.4%) and BC (82.6%) had the highest removal efficiencies. The highest dye uptake was observed at pH 2 with an adsorbent dosage of 0.2 g/100 mL and a dye concentration of 90 mg L^−1^, where the biochar showed an adsorption capacity of 51.28 mg g^−1^. Thus, it can be considered a promising material for environmental remediation. Zhou *et al.* (2018) synthesized an alkali KOH-activated kelp biochar (AKB) and Bi_2_MoO_6_-based composite (BKABC) *via* thermal carbonization and a solvothermal process for dye removal from water.^101^ Up to 324.1 mg g^−1^ methylene blue was adsorbed and 94.12% by AKB, which is attributed to its large surface area (507.177 m^2^ g^−1^), various active sites, and the presence of metal oxides. These results highlighted the capability of AKB to be used as a low-cost, high-efficient adsorbent material in clean water technology and environmental purification. A different study investigated biochar derived from *Ulothrix zonata* macroalgal biomass *via* pyrolysis at 800 °C, revealing its exceptional efficiency in adsorbing dyes such as malachite green, crystal violet, and Congo red. Its enhanced surface area of 133.2 m^2^ g^−1^, thermal stability, and surface chemistry contributed to its maximum adsorption capacity of 5306.2 mg g^−1^ for malachite green, 1222.5 mg g^−1^ for crystal violet, and 345.2 mg g^−1^ for Congo red dye.^102^ This material offers a low-cost, high-performance solution for industrial dye removal while promoting the sustainable utilization of macroalgae waste.

Yao *et al.* (2020) prepared porous biochar from wakame (*Undaria pinnatifida*), a brown alga, *via* a single-step calcination and activation technique and used it for the removal of organic dyes from aqueous environments.^103^ The prepared material possessed a large surface area of 1156.25 m^2^ g^−1^, as well as a mesoporous structure, and consequently it could adsorb organic dyes such as methylene blue (841.64 mg g^−1^), rhodamine B (533.77 mg g^−1^), and malachite green (4066.96 mg g^−1^) very efficiently. The adsorption data best fitted pseudo-second-order kinetics and the Langmuir isotherm. The surface functional groups (–CO, –OH, and –CH) of the material are very active, and they assist both physical and chemical adsorption processes, enabling effective molecular interactions and surface attachments. The entire experiment was carried out as endothermic adsorption, reinforcing the performance of this material and its capability as a low-cost absorbent for wastewater treatment applications. In another study, mesoporous biochar developed from *Eucheuma cottonii* seaweed was used for the efficient removal of methylene blue (MB) dye from synthetic wastewater.^104^ The pyrolysis-produced biochar was subsequently treated with acid, which exhibited improved surface characteristics, including a large surface area (640 m^2^ g^−1^), pore volume, and pore size, all of which contribute to its significant adsorption capacity. The maximum MB adsorption by the synthesized material was 133.33 mg g^−1^ when the conditions were optimized, such as specific MB concentration, pH, and temperature. The adsorption process was chemisorption controlled by monolayer formation on the biochar surface. The main factors that emphasize the possible application of this material in extensive wastewater treatment procedures are its consistent output across a variety of operational parameters and its similar performance to that of commercially available adsorbents.

**Fig. 7:**
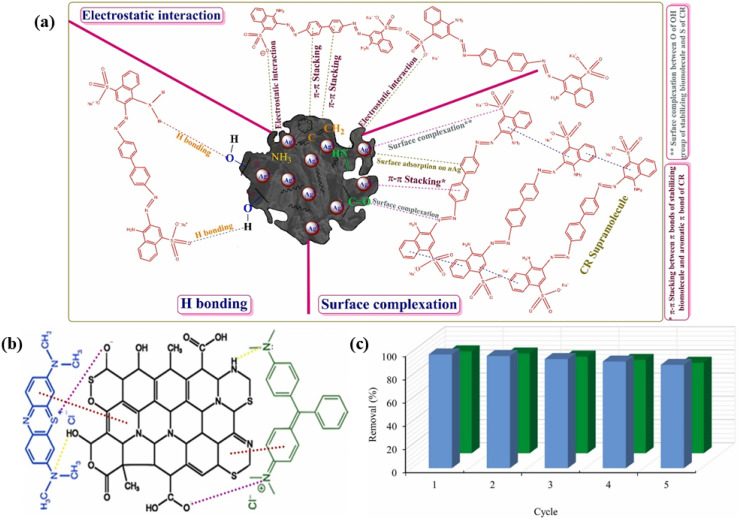
Schematic of (a) CR adsorption *via* mesoporous nAgBC, (b) dye adsorption mechanism through surface functional groups, and (c) efficiency of regeneration across cycles, reproduced from ref. 106 and 109, with permission from ELSEVIER,^106,109^ copyright 2022 and 2023.

In a study by Gurav *et al.* (2021), biochar synthesized from *Eucheuma spinosum* through pyrolysis showed a high adsorption capacity of 331.97 mg g^−1^ for reactive red 120 dye.^105^ The prepared material exhibited a specific surface area of 200.7454 m^2^ g^−1^, and the adsorption data were well described by pseudo-second-order kinetics and the Langmuir isotherm. The removal mechanism indicated that chemisorption was involved through monolayer coverage, and it was mainly driven by electrostatic attractions, ion exchange, metal complexation, and hydrogen bonding, which proved that it can be used as a green adsorption for dye-contaminated water. Moreover, the prepared material retained over 67% of its efficiency after five cycles; hence, it can be reused and is applicable for a long time in the future. Shaikh *et al.* (2022) synthesized a biochar-based silver nanocomposite (nAgBC) derived from *Spirogyra* sp. algal biomass, which demonstrated a high adsorption efficiency for Congo red, achieving 95.92% removal with an adsorption capacity of 34.53 mg g^−1^ under optimal conditions (18 mg L^−1^ of CR, 0.5 g L^−1^ of the absorbent, pH 6, 60 min, and 300 K).^106^ The prepared adsorbent exhibited multilayer chemisorption driven by electrostatic attraction, surface complexation, hydrogen bonding, and van der Waals forces with active groups such as NH, C=O, –OH, SO, and CH, facilitating CR binding, as shown in Fig. 7(a). Reusability tests were also performed, and the results showed that the reuse of this material is feasible. The dye removal efficiency decreased from <8% in the first two cycles to ∼70% by the fifth cycle, which is a great indication of its applications in industrial wastewater treatment.

The research by Wang *et al.* (2022) documented the synthesis of activated carbon from algal bloom biomass, which resulted in a long-lasting and economically attractive adsorbent for the highly efficient removal of dye pollutants from aqueous media.^107^ The resulting material showed a remarkable surface area (2169 m^2^ g^−1^) and adsorption capacity for rhodamine B (1101 ± 11 mg g^−1^), which is much higher than that of rape straw carbon prepared in the laboratory (176 ± 5 mg g^−1^) and commercial activated carbon (489 ± 5 mg g^−1^). The adsorption was driven by both physical and chemical interactions. Physisorption was the dominant adsorption mechanism; however, a minor chemisorption was also present due to π–π interactions and hydrogen bonding between the dye molecule and surface functional groups. The biomass resulting from the pyrolysis of the algal bloom showed flexibility in a wide range of pH and temperature conditions and was able to extract various dyes from natural waters. Therefore, it is necessary to verify its scalability and sustainability for *in situ* wastewater remediation.

In another experiment, the performance of biochar modified with Fe_3_O_4_ was improved to remove methylene blue (MB) as a representative cationic pollutant from water by using biochar prepared from *Ulva fasciata* algae.^108^ The raw and Fe_3_O_4_-integrated biochar revealed very different adsorption capacities of 20.83 and 50.12 mg g^−1^, respectively, with the magnetic sample being able to adsorb more than twice the amount of MB under alkaline conditions and moderate temperature. This enhancement was mainly associated with the more accessible surface area and pore structure brought about by the magnetic modification. The adsorption process was essentially a result of physical interactions, such as pore accommodation and charged-based attractions. However, the process showed reduced spontaneity and molecular disorder besides being thermally favourable. Significantly, the produced biochar revealed great reusability as it maintained 89% removal efficiency over five regeneration cycles, highlighting its practical potential for dye-contaminated water treatment and sustainable pollutant management.

Jafarian *et al.* (2023) synthesized activated biochar from *Sargassum* macro-algae *via* pyrolysis and CO_2_ activation, which showed outstanding adsorption capacities of 500 mg g^−1^ for malachite green and 204.8 mg g^−1^ for methylene blue at pH 7 and temperature of 25 °C.^109^ The adsorption data fitted well with pseudo-second-order kinetics and the Langmuir isotherm. The prepared adsorbent is very porous and has a large surface area (841 m^2^ g^−1^), and is highly efficient in removing malachite green (MG) and methylene blue (MB) dyes from water. Density functional theory simulations revealed that the dye molecules strongly interacted with the functional groups on the adsorbent surface, such as –COONH_2_ and –COOH, which resulted in an increase in adsorption affinity, as shown in Fig. 7(b). After five consecutive regeneration cycles, it was interesting to note that the material still maintained more than 85% removal efficiency for MB and 75% for MG, proving its eco-friendly application for the treatment of dye-contaminated water, as shown in Fig. 7(c). However, in another study, DFT calculations verified a similar mechanism between the biochar-based adsorbent and MB dye.^110,111^ This work showed that the main interactions were primarily governed by functional groups, with the –COOH moieties of the adsorbent forming specific associations with the R–N(CH_3_)_2_ groups of MB. Both studies emphasized that electrostatic interactions, as well as H-bonding interactions, contribute to the adsorption of MB dye. In another investigation, hydrochars obtained *via* the hydrothermal carbonization of *Sargassum muticum* at 180 °C, 240 °C, and 300 °C for 60 min were tested for their capacity to adsorb rhodamine B from aqueous solutions.^112^ The sample heated at 240 °C (SM2) was found to have the highest adsorption capacity of 20.77 mg g^−1^. The samples heated at 300 °C (SM3) and 180 °C (SM1) had adsorption capacities of 17.69 mg g^−1^ and 17.29 mg g^−1^, respectively. Under optimized conditions, SM2 was able to remove 98% of the target within 30 min at 45 °C.

The adsorption process was basically controlled by the chemical interactions of the dye molecules with the active functional groups on the hydrochar surface, which aligns with the use of temperature-treated algal-derived adsorbents as a reliable method for pollutant remediation. Another study also showed the effectiveness of a biochar-sulphur composite material prepared from *Ulva lactuca*, which was chemically modified with H_2_SO_4_ and NaHCO_3_, for the removal of methylene blue (MB) dye from water.^113^ The high equilibrium adsorption capacity of the adsorbent was recorded as 303.78 mg g^−1^, which was basically due to its porous structure and the presence of active surface sites that facilitated both physical and chemical interactions, under optimized experimental conditions with a material dosage of 0.5 g L^−1^ and an initial MB concentration of 200 mg L^−1^. The adsorbent maintained 89.65% of its adsorption capacity for up to six cycles, which shows that it had strong affinity and was very effective in binding dye pollutants in aqueous solutions. In a different investigation, *Sargassum* (brown seaweed), a coastal brown macroalga, was transformed into ultra-porous carbonaceous adsorbents by hydrothermal carbonization (180–260 °C) followed by KOH activation, enabling the efficient removal of methylene blue (MB) dye from wastewater.^114^ The prepared adsorbent showed outstanding physicochemical characteristics, such as a high carbon content, thermal stability, and a broad porosity range with a large surface area ranging from 1216.92–1404.09 m^2^ g^−1^. These structural characteristics, with abundant surface oxygenated acidic groups, made it possible for this material to adsorb methylene blue efficiently with adsorption capacities of 641.03 mg g^−1^ at room temperature and 714.29 mg g^−1^ at a higher temperature (37 °C). The adsorption benefited significantly from a prolonged contact time, alkaline pH, and thermal treatment, indicating temperature-favoured uptake. Pore filling and electrostatic interactions between the dye molecules and the functionalized carbon surface governed the elimination of MB mechanistically. Its ability to selectively bind to MB even in the presence of other dyes, such as methyl orange, demonstrated the potential of this material as an absorbent for targeted pollutant removal in complex wastewater treatment processes. Another study revealed that after the conversion of phycocyanin-extracted algal bloom residues (PE-ABR) into hydrochar *via* hydrothermal carbonization, the hydrochar exhibited a strong adsorption capacity of 89.05 mg g^−1^ for malachite green dye, which is superior to the capacity of unprocessed ABR-derived hydrochar (43.11 mg g^−1^).^115^ The improved adsorption was mostly due to the binding of malachite green molecules with the surface functional groups of the hydrochar, which provides a sustainable and low-energy strategy for the treatment of dye-contaminated wastewater.

Another work presented a groundbreaking strategy for dye removal by creating an algal-based magnetic biochar nanocomposite made from *Ulva fasciata via* carbonization and magnetization, aimed directly at the adsorption of Azocarmine G2 (ACG2), a toxic azo dye.^116^ The synthesized material possessed a surface area of 51.92 m^2^ g^−1^ and efficient adsorption capacity (71.3 mg g^−1^) for ACG2 at pH 1. The adsorption was a result of electrostatic attractions that occurred under acidic conditions, pore-filling in its pore structure, and stabilizing interactions such as π–π electron donor–acceptor attractions, and hydrogen bonding, allowing the dye to be effectively retained on its heterogeneous surface. After five regeneration cycles, the synthesized nanocomposite maintained more than 80% of its adsorption capacity, demonstrating its structural stability and reusability. It also maintained over 90% dye removal effectiveness. These results indicate the potential of this material to be used as a green and efficient adsorbent in real wastewater treatment processes. Recently, a study was carried out on biomass of *sargaço* gathered from the coastlines of Portuguese, in which it was shown that thermal treatment at 400 °C and subsequent ball milling resulted in the production of a very effective biochar for the removal of methylene blue dye.^117^ The prepared biochar showed the maximum adsorption of 500 mg g^−1^, which is not only higher than that of conventional biomass-derived adsorbents but also effectively exhibits π–π interactions, hydrogen bonding, and ion exchange to be the most efficient under alkaline and high-temperature conditions. Research proposed the concept of using invasive macroalgae as a source of new, scalable, environmentally friendly materials for industrial wastewater treatment. In a recent study, hydrochar was prepared from *Spirogyra* sp. algae *via* hydrothermal carbonization to improve its adsorption performance for anionic dyes.^118^ The prepared material exhibited an increased selectivity for direct yellow dye, resulting in 62.07% removal efficiency with a maximum adsorption capacity of 95.24 mg g^−1^ at pH 6. The adsorption process was a naturally occurring one, endothermic, and it was mainly due to the interactions on the surface and the enhanced structural properties. This material also showed excellent reusability, retaining 67.11% efficiency after four cycles, indicating that it can be a stable and environmentally friendly material for the treatment of dye-contaminated water in the future.

Overall, algal transformation into biochar, activated carbon, and hydrochar has proven to be prominent for the removal of dye pollutants from the aquatic environment through various interactions such as H-bonding, electrostatic interactions, and surface complexation. Moreover, chemical modifications and the addition of nanomaterials enhance the efficacy of these materials. These algal-derived materials are promising for the elimination of organic pollutants because of their high biosorption capabilities and reusability.

Table 3 summarizes the reported literature (2016–2025) on algae-derived adsorbents for dye removal. *Ulothrix zonata*- and *Undaria pinnatifida*-derived biochar showed exceptionally high adsorption capacities of 5306.2 and 4066.96 mg g^−1^, respectively, for malachite green dye, outperforming most other algal derived adsorbents. Conversely, hydrochar derived from *Sargassum muticum* demonstrated the lowest adsorption capacity of 20.77 mg g^−1^ for rhodamine B, showing structural limitations compared to biochar. Most studies investigated methylene blue (MB) adsorption in the basic pH range (pH range: 7–12). Because MB is a cationic dye, at low pH, only H^+^ ions are present, which facilitate electrostatic repulsion between MB and the positively charged adsorbent surface, hindering adsorption. In contrast, at high pH, deprotonation of surface functional groups generates negatively charged sites, which promote electrostatic interactions and significantly enhance MB uptake. In general, pyrolysis and chemical activation effectively produce high-quality adsorbents due to their high porosity, enhanced surface area, adsorption capacities, and functional groups. Also, both magnetic enhancement and composite modification enhanced the aspect of reusability and pollutant selectivity through adsorption capacities. Most of the adsorbents were effective in removing a diverse range of dyes, and chemisorption was the predominant mechanism, as indicated by pseudo-second-order kinetics. The Langmuir and Freundlich isotherms are the most reported ones, with Langmuir best fitting monolayer adsorption. Several algae-derived materials and composites retain 75–89% adsorption efficiency after multiple cycles, highlighting their practical applications. In the earlier works (2016–2018), the focus was on basic pyrolysis with moderate capacities, whereas the latest studies (2022–2025) emphasize advanced modifications, including doping, magnetization, and composite fabrication for higher adsorption capacities and reusability.

**Table 3:** Summary of algae-derived adsorbents, highlighting their synthesis methods, dye removal efficiencies, and regeneration performance across multiple cycles

Algal species used	Modification techniques	Prepared adsorbent	Specific surface area (m^2^ g^−1^)	Adsorbed pollutant	Adsorption capacities (mg g^−1^)	Optimized parameters	Adsorption kinetic models and isotherms	Reusability	Year	Ref.
*Spirulina platensis*	Thermal activation	Biochar	167	Congo red	51.28	pH: 2	Freundlich isotherm	—	2016	100
						Adsorbent dosage: 0.2 g/100 mL				
						Temp.: 303 K				
Kelp	Thermal carbonization and solvothermal synthesis	Biochar	507.177	Methylene blue	324.1	pH: not specified	Pseudo-second-order	—	2018	101
						Adsorbent dosage: 0.01 g/50 mL				
						Temp.: not specified				
*Ulothrix zonata*	Pyrolysis	Biochar	133.2	Malachite green	5306.2	pH: 10	Pseudo-second-order and Freundlich isotherm	—	2018	102
						Adsorbent dosage: 0.005 g/10 mL				
						Temp.: 298 K				
*Undaria pinnatifida*	Calcination	Biochar	1156.25	Methylene blue (MB)	841.64	pH: 12 (MB), 12 (RhB), 7 (MG)	Pseudo-second-order and Langmuir isotherm	—	2020	103
				Rhodamine B (RhB)	533.77	Adsorbent dosage: 0.01 g/50 mL				
				Malachite green (MG)	4066.96	Temp.: 323 K				
*Eucheuma cottonii*	Pyrolysis with acid treatment	Biochar	640	Methylene blue	133.33	pH: 4	Pseudo-second-order and Langmuir isotherm	—	2020	104
						Adsorbent dosage: 300 mg/1000 mL				
						Temp.: 348 K				
*Eucheuma spinosum*	Pyrolysis	Biochar	200.7454	Red 120 dye	331.97	pH: 3	Pseudo-second-order and Langmuir isotherm	Retained 67% after five cycles	2021	105
						Adsorbent dosage: 0.025 g/100 mL				
						Temp.: 313 K				
*Spirogyra* sp.	Mild-thermal pyrolysis and co-precipitation method	Ag-biochar nanocomposite	8.77	Congo red	34.53	pH: 6	Pseudo-second-order and Freundlich isotherm	∼75% by the fifth cycle	2022	106
						Adsorbent dosage: 0.5 g/1000 mL				
						Temp.: 300 K				
*Algal bloom*	Pyrolysis	Activated carbon	2169	Rhodamine B	1101 ± 11	pH: 10.80	Pseudo-second-order and Langmuir isotherm	—	2022	107
						Adsorbent dosage: 0.1 g/1000 mL				
						Temp.: 323.15 K				
*Ulva fasciata*	Pyrolysis and magnetic enhancement using ferrous and ferric solutions	Magnetic-biochar	34.5466	Methylene blue	50.12	pH: 9	Pseudo-second-order and Langmuir isotherm	89% over five cycles	2023	108
						Adsorbent dosage: 2 g/1000 mL				
						Temp.: 298 K				
*Sargassum*	Pyrolysis and CO_2_ activation	Activated biochar	841	Malachite green	500	pH: 7	Pseudo-second-order and Langmuir isotherm	75% after five cycles	2023	109
				Methylene blue	204.8	Adsorbent dosage: 0.002 g/5 mL		85% after five cycles		
						Temp.: 298 K				
*Sargassum muticum*	Hydrothermal carbonization	Hydrochar	60.86	Rhodamine B	20.77	pH: not specified	Pseudo-second-order	—	2023	112
						Adsorbent dosage: 30 mg/50 mL				
						Temp.: 513 K				
*Ulva lactuca*	Chemical modification	Biochar-sulphur composite	6.26	Methylene blue	303.78	pH: 12	Pseudo-second-order and Langmuir isotherm	89.65 up to six cycles	2024	113
						Adsorbent dosage: 0.5 g/1000 mL				
						Temp.: 298 K				
*Sargassum*	Hydrothermal carbonization followed by chemical activation	Hydrochar	1404.09	Methylene blue	714.29	pH: 12	Elovich model and Langmuir isotherm	—	2024	114
						Adsorbent dosage: 1 g/1000 mL				
						Temp.: 310 K				
*Ulva fasciata*	Carbonization and magnetization	Magnetic biochar nanocomposite	51.92	Azocarmine G2	71.3	pH: 1	Pseudo-second-order and Langmuir isotherm	More than 80% after five cycles	2025	116
						Adsorbent dosage: 2.5 g/1000 mL				
						Temp.: 298 K				
*Sargaço*	Carbonization followed by ball milling	Biochar	—	Methylene blue	500	pH: 12.6	Langmuir isotherm	—	2025	117
						Adsorbent dosage: 2 g/1000 mL				
						Temp.: 313 K				
*Spirogyra* sp.	Hydrothermal carbonization	Hydrochar	5.369	Direct yellow	95.24	pH: 6	Pseudo-second-order and Freundlich isotherm	67.11% after four cycles	2025	118
						Adsorbent dosage: 0.02 g/20 mL				
						Temp.: 313 K				

### 5.3 Removal of agrochemicals and phenols

Algal-derived adsorbents are also effective for the removal of agrochemicals and phenols. For example, Zheng *et al.* (2017) converted *Chlorella* sp. Cha-01 into biochar through pyrolysis, which showed a high adsorption capacity for *p*-nitrophenol (204.8 mg g^−1^), significantly outperforming the raw biomass and powdered activated carbon.^119^ Its high N/C and O/C ratios, along with a large number of oxygenated functional groups, led to increased polarity and efficient pollutant binding, supporting its use in wastewater remediation. In another experiment, excess *Ulva prolifera* biomass was transformed into N-doped biochar (2.6% nitrogen) through a fast hydrothermal carbonization process for bisphenol A (BPA) removal.^120^ The prepared biochar exhibited a surface area of 25.43 m^2^ g^−1^ and adsorption capacity of 84.19 mg g^−1^ at a high temperature. The uptake of bisphenol A remained consistent with pH and ionic strength, indicating that this material is a reliable candidate for phenolic pollutant remediation. In the study conducted by Cui *et al.* (2019), porous carbon obtained from the biomass of a harmful algal bloom through one-pot carbonization and activation showed the ability to remove phenol effectively, achieving an adsorption capacity of 52 mg g^−1^.^121^ This performance was attributed to its well-developed microporous structure and surface functionalities, underscoring its potential as a sustainable adsorbent for organic pollutant remediation. In recent research, carbonaceous adsorbents made from *Chlorella vulgaris* through HTC and pyrolysis were tested for the removal of metribuzin from water. The reason for the best adsorption performance by the hydrochar at 200 °C was that most of its structure and functional groups remained intact, whereas the pyrochar produced at 500 °C had a larger surface area but lower adsorption capacity. In a different study, Cheng *et al.* (2020) reported that biochar made from *Enteromorpha prolifera* through co-carbonization with FeCl_3_ and ZnCl_2_ had a high surface area (up to 399 m^2^ g^−1^), nitrogen doping, and partial graphitization.^122^ These characteristics made this material capable of adsorbing polycyclic aromatic hydrocarbons from water very effectively (up to 90 mg g^−1^). Adsorption was controlled by pore filling, π–π interactions, mass transfer, and hydrophobic partitioning, with thermal regeneration (∼97% after five cycles) showing its effective reusability in organic pollutant remediation.

In another study, a magnetic biochar/sulfidated Fe^0^ composite (S-Fe^0^/BC) was prepared through a green one-step method using *Ulva prolifera*.^123^ In this process, the composite managed to remove 88% removal of tetrabromobisphenol A (TBBPA), governed by chemical adsorption, reductive debromination, and electron transfer. This material possessed a surface area of 47.2 m^2^ g^−1^ and a stable performance with a capacity of 1.47 mg g^−1^, retaining more than 1.17 mg g^−1^ after six regeneration cycles. Furthermore, the adsorption data aligned well with pseudo-second-order kinetics and the Freundlich isotherm. Vinayagam *et al.* (2023) reported further improvements in this area by developing a magnetic biochar composite (UPAC–Fe_2_O_3_) through chemical activation, thermal carbonization, and *in situ* co-precipitation of ferric oxide onto *Ulva prolifera*-based activated carbon, which effectively removed the herbicide 2,4-dichlorophenoxyacetic acid (2,4-D) from water through surface-mediated interactions.^124^ Its mesoporous structure and large surface area (292.51 m^2^ g^−1^) supported pollutant adsorption through mechanisms such as hydrogen bonding, electrostatic attraction, and chemical affinity between functional groups and the herbicide molecules. The composite achieved an adsorption capacity of 60.61 mg g^−1^, demonstrating its feasibility for removing organic pollutants from water. In addition, pseudo-second-order kinetics and the Langmuir isotherm best fitted the adsorption data.

Song *et al.* (2024) developed nitrogen-enriched porous biochars (NPB) through co-pyrolysis of *Enteromorpha prolifera* (E-NPB), *Ulva lactuca* (U-NPB), and oyster shell, which showed a high surface area of 1501.80 m^2^ g^−1^ for E-NPB and 1067.18 m^2^ g^−1^ for U-NPB and significant pore volumes, resulting in the efficient adsorption of atrazine (312.06 and 340.52 mg g^−1^), respectively.^125^ Hydrogen-bonding, pore filling, π–π interactions, and electrostatic interactions controlled the multilayer sorption process. It is worthwhile noting that the biochars still maintained significant adsorption capacities (246.13 and 255.97 mg g^−1^, respectively) after they were reused twice, which indicated their possible applications as green materials for water purification using algal-derived adsorbents. Moreover, the adsorption data best fitted pseudo-second-order kinetics and the Freundlich isotherm. Yu *et al.* (2022) prepared iron-modified biochar from *Aegagropila linnaei*, and showed that the bisphenol A removal efficiency of 69.8% ± 2.3% at pH 3, which was attributed to the high surface area (144.62 m^2^ g^−1^), increased porosity, and electron conductivity of this material.^126^ The surface interactions and reactive oxygen species generated by nano-Fe_3_O_4_ under acidic conditions mainly controlled the adsorption, providing a cheap and environmentally friendly way to help reduce bisphenol-A contamination in water bodies.

In our opinion, the use of algae-derived adsorbents provides a green and flexible approach for the elimination of organic pollutants. Their adsorption capacity, stability, and reusability were consistently improved by modifying their structural using various methods, metal doping, and material combinations. These materials are quite successful and may be employed in large-scale water cleanup operations, even if their adsorption mechanism varies throughout the literature. We believe that their environmentally friendly nature positions them as promising materials for next-generation water treatment technologies.

Table 4 highlights the use of adsorbents produced from algae for the removal of agrochemicals. Early pyrolysis/hydrothermal carbons had modest surface areas and moderate capacities, but more recent doped/activated biochars obtained specific surface areas greater than 1500 m^2^ g^−1^ and adsorption capacities of more than 300 mg g^−1^. Algal adsorbents vary greatly, and they are dominated by pseudo-second-order kinetics with the Freundlich/Langmuir isotherms. In subsequent research, their reusability increased, demonstrating the enhanced efficiency, pollutant selectivity, and potential for sustained regeneration of doped biochars.

**Table 4:** Key parameters of algal-based adsorbents in the context of synthesis, pollutant removal, and reusability for agrochemicals and phenols

Algal species used	Synthesis techniques	Prepared adsorbent	Specific surface area (m^2^ g^−1^)	Adsorbed pollutant	Adsorption capacities (mg g^−1^)	Optimized parameters	Adsorption kinetic models and isotherms	Reusability	Year	Ref.
*Chlorella* sp. Cha-01	Pyrolysis	Biochar	6.163	*p*-Nitrophenol	204.8	pH: 11	Pseudo-second-order and Freundlich isotherm	—	2017	119
						Adsorbent dosage: 0.02 g/20 mL				
						Temp.: 303.15 K				
*Ulva prolifera*	Rapid hydrothermal carbonization	N-doped biochar	25.43	Bisphenol A	84.19 under elevated temperature	pH: 7–10	Pseudo-second-order and Langmuir isotherm	—	2017	120
						Adsorbent dosage: 10 mg/10 mL				
						Temp.: 318 K				
Harmful algal bloom biomass	Carbonization and activation	Porous carbon	430	Phenol	52	pH: 5	Pseudo-second-order and Langmuir isotherm	—	2019	121
						Adsorbent dosage: 0.015 g/15 mL				
						Temp.: room temperature				
*Enteromorpha prolifera*	Co-carbonization with FeCl_3_ and ZnCl_2_	Biochar	57	Polycyclic aromatic hydrocarbons	90 for naphthalene	pH: 6	Pseudo-second-order, intra particle diffusion and Freundlich isotherm	∼97% after five cycles	2020	122
						Adsorbent dosage: 0.001 g/20 mL				
						Temp.: 298 K				
*Ulva prolifera*	Hydrothermal and sulfidation	Biochar	47.2	Tetrabromobisphenol A	1.47	pH: 4	Pseudo-second-order and Freundlich isotherm	Retained over 1.17 mg g^−1^ after six regeneration cycles	2020	123
						Adsorbent dosage: 0.6 g/1000 mL				
						Temp.: 308				
*Ulva prolifera*	Chemical activation, thermal carbonization, and co-precipitation	Activate carbon	292.51	Dichlorophenoxyacetic acid	60.61	pH: 2	Pseudo-second-order and Langmuir	—	2023	124
						Adsorbent dosage: 2 g/1000 mL				
						Temp.: 303 K				
*Enteromorpha prolifera*, *Ulva lactuca*	Co-pyrolysis	Nitrogen-doped biochars	1501.80 for (E-NPB) and 1067.18 for (U-NPB)	Atrazine	312.06 for (E-NPB)	pH range: 6–7	Pseudo-second-order and Freundlich isotherm	Retained 246.13 mg g^−1^ for (E-NPB), 255.97 mg g^−1^ for U-NPB	2024	125
					340.52 for U-NPB	Adsorbent dosage: 0.001 g/20 mL				
						Temp.: 298 K				
Microalgae	Hydrothermal carbonization	Hydrochar	15.3	Bisphenol A	25.8	pH: 7	Langmuir isotherm	—	2025	99
						Adsorbent dosage: 0.05 g/50 mL				
						Temp.: 293 K				

## 6. Influencing factors and reusability analysis

### 6.1 Influencing factors

The adsorption capacities of carbon-rich materials are strongly dependent on their pore size, surface area, and particle size, where hierarchical porosity and optimized distribution facilitate active site accessibility, thereby allowing the effective removal of diverse pollutants and their application as advanced water treatment technologies.^127^ The adsorption of pollutants onto carbon-rich materials is facilitated by various mechanisms such as electrostatic interactions, hydrogen bonding, precipitation, and complexation, where high porosity and oxygen-rich functional groups are present to facilitate these interactions.^128,129^

**Fig. 8:**
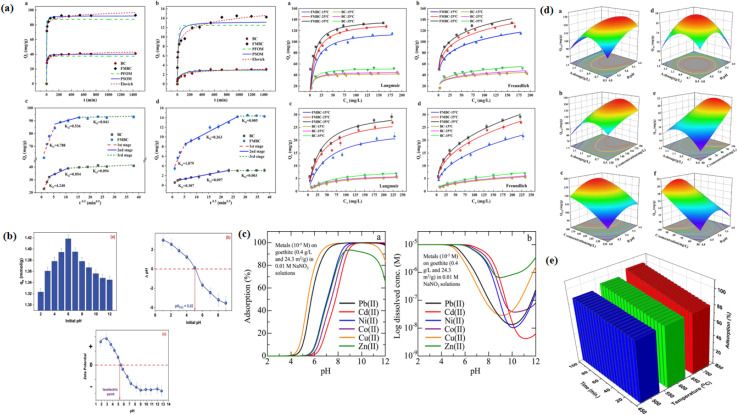
Environmental factors, including (a) kinetic adsorption model for Cd(ii) and As(iii), reproduced from ref. 129, with permission from ELSEVIER,^129^ copyright 2023. (b) Influence of pH, reproduced from ref. 131, with permission from Taylor & Francis,^131^ copyright 2026. (c) Influence of pH, reproduced from ref. 132, with permission from ELSEVIER,^132^ copyright 2024. (d) Interaction effects of various factors, reproduced from ref. 129, with permission from ELSEVIER,^129^ copyright 2023 and (e) influence of calcination temperature, reproduced from ref. 131, with permission from Taylor & Francis,^131^ copyright 2026.

Adsorption efficiency depends on factors such as contaminant solubility, charge interactions, solution pH, temperature, ionic strength, contact time, and mixing intensity, which determine the form of pollutants in water, their interactions with the adsorbent surface, and molecular movements, ultimately deciding the adsorption process.^130–133^ For example the removal efficiency of methyl orange is influenced by pH, concentration, and contact time, with the optimal adsorption observed at pH 6, 220 ppm, and 180 min, where physically activated carbon demonstrated the highest removal capacity (∼99%), outperforming raw algae and chemically activated and commercial carbons, highlighting its excellent surface properties and interaction capabilities under controlled conditions.^134^ Similarly, *Spirulina*-derived activated carbon showed high adsorption capacities of 660.5 mg g^−1^ for metoprolol and 588.9 mg g^−1^ for diclofenac at 25 °C and pH 5.0 ± 1.0, in addition to removing over 88% of 17 out of 20 micropollutants spiked at 100 µg L^−1^ in complex wastewater.^135^ In a different investigation, it was reported that the adsorption efficiency of *Spirulina platensis* and commercial activated carbon for reactive red 120 dye was primarily affected by pH, contact time, and temperature.^136^ The highest adsorption was observed at pH 2 and temperature of 298 K, where the protonation of surface functional groups enhanced the electrostatic interactions with the anionic dye species. A longer contact time made it possible for the dye molecules and the active sites to interact; thus, equilibrium was attained, and the total adsorption capacity was increased. These results support the adaptability and efficacy of designed bio-based adsorbents across several contaminant classes by showing that the intensity of contact and the uptake dynamics are determined by pH-dependent speciation, pollutant concentration, and ambient conditions. Developing adsorbents that are effective, selective, and eco-friendly requires a thorough grasp of the adsorption principles, adsorption influencing parameters, and performance metrics. This will enable the removal of contaminants from water systems at a reduced cost. The impact factors are shown schematically in Fig. 8.

### 6.2 Regeneration and reusability

Regenerability is one of the most important factors in determining the efficiency of an adsorbent, as it allows for the economically viable and environmentally friendly reuse of exhausted materials by technically feasible recovery methods, hence increasing the implementation potential of adsorption for the removal of contaminants from water.^137^ Recycling adsorbents helps to maintain economic sustainability as they can be used for several reuse cycles with a slight loss of performance. However, their long-term use might result in decreased efficiency caused by incomplete desorption, degradation of active sites, or changes in surface characteristics and porosity owing to consecutive regeneration cycles.^138^ In the case of organic pollutants such as dyes and pharmaceuticals, the regeneration agents are usually solvents, including methanol, ethanol, and acetone, which allow effective desorption and repeated use in water environments.^139^ It is very important that the used adsorbents are disposed of or recycled in a safe way, because if they are not properly managed, they may cause secondary pollution, especially in areas that do not have incineration or landfilling facilities, and thorough desorption and toxicity evaluation is required.^140^

**Fig. 9:**
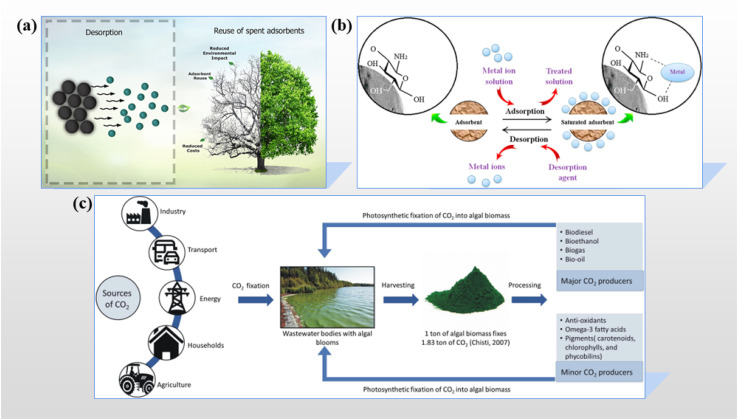
Schematic of regeneration and reusability, including (a) reuse of spent adsorbents, reproduced from ref. 138, with permission from ELSEVIER,^138^ copyright 2022. (b) Schematic of the regeneration procedure, reproduced from ref. 137, with permission from ELSEVIER,^137^ copyright 2019. (c) Schematic of carbon neutral nature, reproduced from ref. 141, with permission from ELSEVIER,^141^ copyright 2020.

Regardless of their impressive regeneration performance, as discussed in Tables 2 and 3, stability over a long period of time under changing environmental conditions and the fate of residual contaminants are aspects that need to be clarified further. Future studies should focus on optimizing regeneration processes and toxicological evaluation of desorption so that reusing or throwing away can be carried out without any risk. A schematic diagram of regeneration and reusability is shown in Fig. 9.

## 7. Environmental, economic, and LCA considerations

Algal-derived adsorbents are highly beneficial to the environment and economically viable for the removal of organic pollutants, as they are renewable, have a low production cost, and have a low impact on the environment, thus representing a sustainable alternative to the use of conventional adsorbents in the treatment of wastewater. To quantify both ecological performance and cost effectiveness, algal-derived adsorbents can be evaluated through the use of the LCA (life cycle assessment) and LCCA (life cycle cost analysis) methodologies. LCA and LCCA are complementary methods for evaluating renewable and biomass-based systems. LCA, according to ISO 14040, thoroughly identifies the environmental impacts across the entire supply chain, including climate change and ozone depletion.^142^ Alternatively, LCCA measures the overall financial performance of a product or system over a period of time, considering the purchase, operational maintenance, and disposal cost. LCA and LCCA together offer a comprehensive platform for achieving environmental sustainability and economic feasibility of technologies such as biochar and bioenergy production.

Wang *et al.* (2023) documented the potential of algae-based adsorbents in removing tetracycline.^143^ They showed that co-pyrolysis of *Enteromorpha* (EN) and *Chlorella vulgaris* (CV) at 500 °C and a 2 : 8 ratio, and NaOH activation at 800 °C achieved a removal efficiency of 97.56%. Further LCA using open LCA showed that the production of the EN-based adsorbent had the least environmental impact, mainly because its ocean cultivation requires significantly less freshwater compared to the freshwater-intensive cultivation of CV. Moreover, sensitivity analysis illustrated that the reuse of wastewater instead of aquaculture water could drastically lower the environmental load of the mixed algae systems, thus making them more eco-friendly. These results highlighted that even though adsorbents derived from algae are very potent, the carbon footprints of these materials are heavily dependent on their mode of cultivation, therefore providing a road map for the future preparation and applications of these materials.

Extending this sustainability perspective beyond pollutant removal, the contribution to the environment becomes even more significant when considering that algae is a carbon-neutral source. They can be grown on non-arable land or in nutrient-rich wastewater, enabling simultaneous nutrient recovery and carbon-dioxide sequestration during their growth. In this respect, microalgae, especially through their high photosynthetic efficiency, which is 40–50% higher than that of terrestrial plants, and their ability to capture 1.83 kg of CO_2_ per kilogram of biomass, significantly contribute to environmental remediation.^141,144,145^ They complete the nitrogen and phosphorus uptake as well and serve as bioindicators, supporting aquatic monitoring and the generation of valuable biomass. After the biomass is harvested, its conversion into hydrochar, biochar, and activated carbon allows for waste valorization and the lowering of the use of fossil-based adsorbents, therefore promoting the principles of a circular economy in eco-friendly pollutant management.^59^

**Fig. 10:**
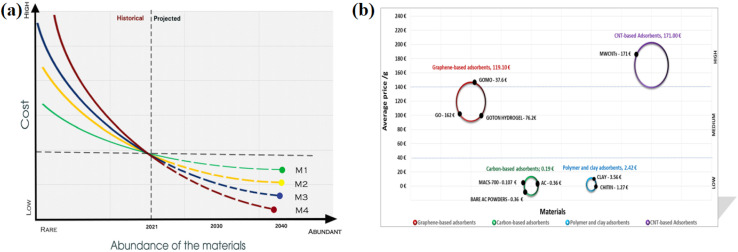
Environmental economics analysis, including (a) breakdown of average cost per material group and (b) declining curves depicting cost as a function of the availability of various materials. Each of the materials (M1, M2, M3, and M4) represents a different level of cost, reproduced from ref. 138, with permission from ELSEVIER,^138^ copyright 2022.

However, additional adjustments are required to properly deal with energy and post-treatment costs. Accordingly, this research focuses on the adsorption properties and the post-treatment conversion of algae-based materials, evaluates their removal efficiency, and their general applicability as a means of developing environmentally friendly wastewater treatment methods. A schematic diagram of the environmental economics analysis is shown in Fig. 10.

## 8. Future prospective and research gaps

**Fig. 11:**
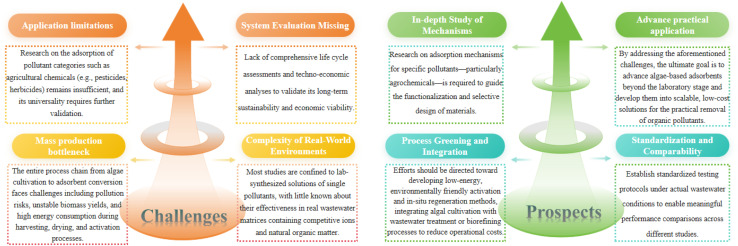
Schematic of current challenges and future perspectives.

Although algae-derived adsorbents have shown great potential for removing organic pollutants such as dyes and pharmaceuticals, their large-scale application remains limited by several challenges. Particularly, their use for the removal of agrochemical contaminants such as pesticides, fertilizers, and herbicides is still underexplored. These limitations emphasize that they need to be validated broadly over different pollutant classes and under various operational conditions, with further support from DFT/computational studies to enable the design of eco-friendly water treatment systems. The majority of studies are limited to controlled laboratory environments where synthetic solutions are employed, providing little understanding of the effectiveness in complex wastewater matrices that contain competing ions and natural organic matter. Challenges include contamination risks, inconsistent biomass yields, and high energy demands associated with harvesting, drying, and activation. Additionally, scaling up algal cultivation exacerbates these energy demands. Further, the conversion of algal biomass into biochar, hydrochar, or activated carbon requires energy-intensive processes that increase the cost of production. Next, researchers need to emphasize the development of energy-efficient and environment-friendly methods for activating on-site regeneration, and integrated processes that allow algal growth in wastewater treatment or biorefinery operations to lower the running costs to overcome these obstacles. To improve the surface functionality and selectivity, it is necessary to conduct detailed studies on the adsorption mechanism, specifically targeting agrochemicals. Besides, to determine the sustainability of a material in the long term, a thorough life cycle and techno-economic assessment should be performed. Additionally, standardized testing under real conditions will make it possible to compare different studies better. Bridging these gaps will help the development of algae-based adsorbents to go beyond the lab experiment stage to become scalable, affordable solutions for the removal of organic pollutants. A schematic diagram of the current challenges and future perspectives is shown in Fig. 11.

## 9. Conclusions

Algal-derived adsorbents demonstrate exceptional flexibility and efficiency in sequestering an extensive range of organic pollutants, such as dyes, pharmaceuticals, and agrochemicals. Their adsorption capacities differ substantially, ranging from 20 to more than 5300 mg g^−1^, depending on the method and the contaminant to be removed. By using different thermal and chemical methods such as pyrolysis, hydrothermal carbonization, and chemical activation, algal biomass can be converted into biochar, hydrochar, and activated carbon, which possess enhanced surface areas, porosity, and functional groups. These materials adsorb pollutants by various mechanisms such as π–π interactions, electrostatic interactions, ion exchange, and hydrogen bonding. Most of these materials maintained 70–95% removal efficiency through several regeneration cycles, and thus they can be considered highly reusable. However, large-scale cultivation, biomass processing, and energy-consuming activation are still the key limitations. Future studies need to be conducted on energy-efficient, green synthesis methods, targeted agrochemical adsorption studies, and pilot-scale validation under real wastewater conditions. In general, carbon materials sourced from algae represent a green and circular supply for advanced water treatment and pollutant control.

## Author contributions

Tongtong Wang: methodology, conceptualization, data curation, formal analysis, funding acquisition, project administration, writing-original draft, and writing-review & editing. Soni Patyal: investigation, data curation, formal analysis, writing-original draft. Mu. Naushad, Pooja Dhiman, Sen Wang, Weiqian Li, and Jinjun Cai: writing-review & editing. Gaurav Sharma: resources, writing-review & editing. Ying Gao: visualization and investigation.

## Conflicts of interest

The authors declare that they have no known competing financial interests or personal relationships that could have appeared to influence the work reported in this paper.

## Data Availability

Data sharing is not applicable to this article as no new data were generated or analyzed in this study.

## References

[cit1] Ramesh B. (2023). *et al.*, A review on algae biosorption for the removal of hazardous pollutants from wastewater: Limiting factors, prospects and recommendations. Environ. Pollut..

[cit2] Okoro H. K. (2022). *et al.*, Nanomaterial-based biosorbents: Adsorbent for efficient removal of selected organic pollutants from industrial wastewater. Emerging Contam..

[cit3] Bhatt P. (2022). *et al.*, Algae in wastewater treatment, mechanism, and application of biomass for production of value-added product. Environ. Pollut..

[cit4] Abdalla Y. , *et al.*, Sustainable Algae Applications in Wastewater Treatment, in Comprehensive Green Materials, ed. P. A. G. Olabi, Elsevier, Oxford, 1st edn, 2025, pp. 82–92

[cit5] Soffian M. S. (2022). *et al.*, Carbon-based material derived from biomass waste for wastewater treatment. Environ. Adv..

[cit6] Satpati G. G. (2024). *et al.*, Synthesis, delineation and technological advancements of algae biochar for sustainable remediation of the emerging pollutants from wastewater-a review. Environ. Res..

[cit7] Arora N. (2024). *et al.*, Algal-based biochar and hydrochar: A holistic and sustainable approach to wastewater treatment. Chem. Eng. J..

[cit8] Ufarté L. (2015). *et al.*, Metagenomics for the discovery of pollutant degrading enzymes. Biotechnol. Adv..

[cit9] Bessegato G. G., Brugnera M. F., Zanoni M. V. B. (2019). Electroanalytical sensing of dyes and colorants. Curr. Opin. Electrochem..

[cit10] Tkaczyk A., Mitrowska K., Posyniak A. (2020). Synthetic organic dyes as contaminants of the aquatic environment and their implications for ecosystems: A review. Sci. Total Environ..

[cit11] Kant R. (2012). Textile dyeing industry an environmental hazard. Nat. Sci..

[cit12] Puzyn T. and Mostrag A., Organic Pollutants Ten Years after the Stockholm convention: Environmental and Analytical Update, BoD – Books on Demand, 2012

[cit13] Din M. I. (2021). *et al.*, Fundamentals and photocatalysis of methylene blue dye using various nanocatalytic assemblies- a critical review. J. Cleaner Prod..

[cit14] Aragaw T. A. (2024). A review of dye biodegradation in textile wastewater, challenges due to wastewater characteristics, and the potential of alkaliphiles. J. Hazard. Mater. Adv..

[cit15] Rawat D., Mishra V., Sharma R. S. (2016). Detoxification of azo dyes in the context of environmental processes. Chemosphere.

[cit16] Mandal A. , *et al.*, Chapter 7 – Impact of agrochemicals on soil health, in Agrochemicals Detection, Treatment and Remediation, ed. M. N. V. Prasad, Butterworth-Heinemann, 2020, pp. 161–187

[cit17] Sarkar B. , *et al.*, Chapter 8 – Sorption and desorption of agro-pesticides in soils, in Agrochemicals Detection, Treatment and Remediation, ed. M. N. V. Prasad, Butterworth-Heinemann, 2020, pp. 189–205

[cit18] Sharma A. (2019). *et al.*, Worldwide pesticide usage and its impacts on ecosystem. SN Appl. Sci..

[cit19] Elumalai P. (2025). *et al.*, Agrochemical pollution: A serious threat to environmental health. Current Opinion in Environmental Science & Health.

[cit20] Singh D. , *et al.*, Chapter 4 – Impacts of agrochemicals on soil microbiology and food quality, in Agrochemicals Detection, Treatment and Remediation, ed. M. N. V. Prasad, Butterworth-Heinemann, 2020, pp. 101–116

[cit21] Dong X. (2024). *et al.*, Mechanisms of adsorption and functionalization of biochar for pesticides: A review. Ecotoxicol. Environ. Saf..

[cit22] Ranjan N., Singh P. K., Maurya N. S. (2022). Pharmaceuticals in water as emerging pollutants for river health: A critical review under Indian conditions. Ecotoxicol. Environ. Saf..

[cit23] Khalid M., Abdollahi M. (2021). Environmental Distribution of Personal Care Products and Their Effects on Human Health. Iran. J. Pharm. Res..

[cit24] Gao Y. (2025). *et al.*, Potential risks and hazards posed by the pressure of pharmaceuticals and personal care products on water treatment plants. Environ. Pollut..

[cit25] Xie W. Y., Shen Q., Zhao F. J. (2018). Antibiotics and antibiotic resistance from animal manures to soil: a review. Eur. J. Soil Sci..

[cit26] Panigrahy N. (2022). *et al.*, A comprehensive review on eco-toxicity and biodegradation of phenolics: Recent progress and future outlook. Environ. Technol. Innovation.

[cit27] Stephen D., Bakthavatsalam A. K. (2019). A comparative study on growth and degradation behavior of C. pyrenoidosa on synthetic phenol and phenolic wastewater of a coal gasification plant. J. Environ. Chem. Eng..

[cit28] Ji Q. (2016). *et al.*, A review on the coal gasification wastewater treatment technologies: past, present and future outlook. J. Cleaner Prod..

[cit29] Wang W. (2011). *et al.*, Treatment of coal gasification wastewater by a two-continuous UASB system with step-feed for COD and phenols removal. Bioresour. Technol..

[cit30] Gaur V. K., Gupta S., Pandey A. (2022). Evolution in mitigation approaches for petroleum oil-polluted environment: recent advances and future directions. Environ. Sci. Pollut. Res..

[cit31] Ahmed F., Fakhruddin A. N. M. (2018). A review on environmental contamination of petroleum hydrocarbons and its biodegradation. Int. J. Environ. Sci. Nat. Res..

[cit32] Sharma B., Dangi A. K., Shukla P. (2018). Contemporary enzyme based technologies for bioremediation: A review. J. Environ. Manage..

[cit33] Dua M. (2002). *et al.*, Biotechnology and bioremediation: successes and limitations. Appl. Microbiol. Biotechnol..

[cit34] Shetty S. S. (2023). *et al.*, Environmental pollutants and their effects on human health. Heliyon.

[cit35] Pearson J. C. (2025). *et al.*, Tetracyclines, the old and the new: A narrative review. CMI Communications.

[cit36] Gopinathan R., Kanhere J., Banerjee J. (2015). Effect of malachite green toxicity on non target soil organisms. Chemosphere.

[cit37] Lee K. C., Wu J. L., Cai Z. (2006). Determination of malachite green and leucomalachite green in edible goldfish muscle by liquid chromatography–ion trap mass spectrometry. J. Chromatogr. B.

[cit38] Culp S. J. (1999). *et al.*, Toxicity and metabolism of malachite green and leucomalachite green during short-term feeding to Fischer 344 rats and B6C3F1 mice. Chem.-Biol. Interact..

[cit39] Cha C. J., Doerge Daniel R., Cerniglia Carl E. (2001). Biotransformation of Malachite Green by the FungusCunninghamella elegans. Appl. Environ. Microbiol..

[cit40] Mitrowska K., Posyniak A., Zmudzki J. (2007). The effects of cooking on residues of malachite green and leucomalachite green in carp muscles. Anal. Chim. Acta.

[cit41] Siddiqui S. I. (2023). *et al.*, Investigation of Congo Red Toxicity towards Different Living Organisms: A Review. Processes.

[cit42] Verma Y. (2025). *et al.*, Chitin-cl-poly (acrylamide-co-guar gum) – Nickel oxide-Zirconium phosphate nanocomposite hydrogel as miniature adsorptional-photocatalysts units for removal of Tetracycline. Inorg. Chem. Commun..

[cit43] Ko Y. G. (2024). Hybrid method integrating adsorption and chemical precipitation of heavy metal ions on polymeric fiber surfaces for highly efficient water purification. Chemosphere.

[cit44] Ossai I. C. (2026). *et al.*, Progress in wastewater treatment, separation and purification technologies: mechanisms, benefits, challenges, efficiencies, and sustainability perspectives. J. Environ. Manage..

[cit45] Mao N. , Nonwoven fabric filters, in Advances in Technical Nonwovens, G. Kellie, Woodhead Publishing, 2016, pp. 273–310

[cit46] Singh N. B. (2018). *et al.*, Water purification by using Adsorbents: A Review. Environ. Technol. Innovation.

[cit47] Mazur L. P. (2018). *et al.*, Brown marine macroalgae as natural cation exchangers for toxic metal removal from industrial wastewaters: A review. J. Environ. Manage..

[cit48] Yargeau V. , Water and wastewater treatment: chemical processes, in Metropolitan Sustainability, ed. F. Zeman, Woodhead Publishing, 2012, pp. 390–405

[cit49] Lu H. (2017). *et al.*, Crystallization techniques in wastewater treatment: An overview of applications. Chemosphere.

[cit50] Saravanan A. (2022). *et al.*, A detailed review on advanced oxidation process in treatment of wastewater: Mechanism, challenges and future outlook. Chemosphere.

[cit51] Gao W. (2025). *et al.*, Comprehensive assessment of membrane technology for typical water treatment processes: A critical review. Desalination.

[cit52] Aziz S. (2024). *et al.*, A comprehensive review of membrane-based water filtration techniques. Appl. Water Sci..

[cit53] Gahlot S. and Kulshrestha V., Fluoropolymer nanocomposite membranes for fuel cell applications, in Advanced Fluoropolymer Nanocomposites, ed. K. Deshmukh and C. M. Hussain, Woodhead Publishing, 2023, pp. 597–643

[cit54] Mondal S., Cho S., Lee J. (2025). Material innovations and challenges in adsorption-based nanoplastic removal for water purification: A comprehensive review of efficacy, mechanisms, performance factors, and future perspectives. J. Environ. Chem. Eng..

[cit55] El Meziani S. (2025). *et al.*, Sustainable adsorption technologies for textile dye removal: Advances in biomass-derived and magnetically modified activated carbons. Cleaner Chemical Engineering.

[cit56] Akhtar M. S., Ali S., Zaman W. (2024). Innovative Adsorbents for Pollutant Removal: Exploring the Latest Research and Applications. Molecules.

[cit57] Satyam S., Patra S. (2024). Innovations and challenges in adsorption-based wastewater remediation: A comprehensive review. Heliyon.

[cit58] Basic Characteristics of the Algae, in Phycology, ed. R. E. Lee, Cambridge University Press, Cambridge, 2018, pp. 2–30

[cit59] González Fernández L. A. (2025). *et al.*, Algal-Based Carbonaceous Materials for Environmental Remediation: Advances in Wastewater Treatment, Carbon Sequestration, and Biofuel Applications. Processes.

[cit60] Mishra H. N. , Mazumder A. and Prabuthas P., Recent Developments on Algae as a Nutritional Supplement, in Algal Biorefinery: An Integrated Approach, ed. D. Das, Springer International Publishing, Cham, pp. 219–233

[cit61] Maghimaa M. (2025). *et al.*, Exploring algal diversity for enhanced nutrition: Implications for human health and sustainability. Algal Res..

[cit62] Ahmed N. (2024). *et al.*, Comprehensive exploration of marine algae diversity, bioactive compounds, health benefits, regulatory issues, and food and drug applications. Measurement: Food.

[cit63] Dayana Priyadharshini S. (2021). *et al.*, Phycoremediation of wastewater for pollutant removal: A green approach to environmental protection and long-term remediation. Environ. Pollut..

[cit64] Sarıtaş S. (2024). *et al.*, Biological and nutritional applications of microalgae. Nutrients.

[cit65] Cheng L. (2006). *et al.*, Carbon dioxide removal from air by microalgae cultured in a membrane-photobioreactor. Sep. Purif. Technol..

[cit66] Zhou W. (2017). *et al.*, Bio-mitigation of carbon dioxide using microalgal systems: Advances and perspectives. Renew. Sustain. Energy Rev..

[cit67] Hernández A. (2024). *et al.*, Review microalgae drying: A comprehensive exploration from conventional air drying to microwave drying methods. Future Foods.

[cit68] Wang C. (2024). *et al.*, Research advances on production and application of algal biochar in environmental remediation. Environ. Pollut..

[cit69] Luo Y. (2023). *et al.*, Conversion of waste plastics into value-added carbon materials. Environ. Chem. Lett..

[cit70] de Morais E. G. (2023). *et al.*, Biomass valorization via pyrolysis in microalgae-based wastewater treatment: Challenges and opportunities for a circular bioeconomy. J. Appl. Phycol..

[cit71] Tsarpali M., Kuhn J. N., Philippidis G. P. (2024). Activated carbon production from algal biochar: Chemical activation and feasibility analysis. Fuel Commun..

[cit72] Ao W. (2018). *et al.*, Microwave assisted preparation of activated carbon from biomass: A review. Renew. Sustain. Energy Rev..

[cit73] Broch A. (2014). *et al.*, Analysis of Solid and Aqueous Phase Products from Hydrothermal Carbonization of Whole and Lipid-Extracted Algae. Energies.

[cit74] Tsarpali M., Kuhn J. N., Philippidis G.
P. (2022). Hydrothermal Carbonization of Residual Algal Biomass for Production of Hydrochar as a Biobased Metal Adsorbent. Sustainability.

[cit75] Yang Y. (2026). *et al.*, Biomass-derived hydrothermal carbonization carbon: a carbon-negative photocatalytic platform. Bioresour. Technol..

[cit76] Khan A. A. (2025). *et al.*, Algal biochar: A natural solution for the removal of Congo red dye from textile wastewater. J. Taiwan Inst. Chem. Eng..

[cit77] Zhang L. (2023). *et al.*, The Effect of Nitrogen- and Oxygen-Containing Functional Groups on C_2_H_6_/SO_2_/NO Adsorption: A Density Functional Theory Study. Energies.

[cit78] Murtaza G. (2022). *et al.*, A review of mechanism and adsorption capacities of biochar-based engineered composites for removing aquatic pollutants from contaminated water. Front. Environ. Sci..

[cit79] Li L. (2025). *et al.*, Production of Algae-Derived Biochar and Its Application in Pollutants Adsorption—A Mini Review. Separations.

[cit80] Luo Z. (2022). *et al.*, Novel insights into the adsorption of organic contaminants by biochar: A review. Chemosphere.

[cit81] Suresh R. (2024). *et al.*, A review on algae-mediated adsorption and catalytic processes for organic water pollution remediation. Front. Mater..

[cit82] González Fernández L. A. (2025). *et al.*, Algal-Based Carbonaceous Materials for Environmental Remediation: Advances in Wastewater Treatment, Carbon Sequestration, and Biofuel Applications. Processes.

[cit83] Peng L. (2014). *et al.*, Iron improving bio-char derived from microalgae on removal of tetracycline from aqueous system. Environ. Sci. Pollut. Res..

[cit84] Ali M. E. M. (2018). *et al.*, Removal of pharmaceutical pollutants from synthetic wastewater using chemically modified biomass of green alga Scenedesmus obliquus. Ecotoxicol. Environ. Saf..

[cit85] Ouasfi N. (2019). *et al.*, Carbonaceous material prepared by ultrasonic assisted pyrolysis from algae (Bifurcaria bifurcata): Response surface modeling of aspirin removal. Surf. Interfaces.

[cit86] Ouasfi N. (2019). *et al.*, Selected pharmaceuticals removal using algae derived porous carbon: experimental, modeling and DFT theoretical insights. RSC Adv..

[cit87] Choi Y.-K. (2020). *et al.*, Adsorption behavior of tetracycline onto Spirulina sp. (microalgae)-derived biochars produced at different temperatures. Sci. Total Environ..

[cit88] Atugoda T. (2021). *et al.*, Mechanistic interaction of ciprofloxacin on zeolite modified seaweed (Sargassum crassifolium) derived biochar: Kinetics, isotherm and thermodynamics. Chemosphere.

[cit89] Chen L. (2024). *et al.*, A three-step process to produce biochar with good magnetism, high specific surface area, and high levels of nitrogen doping for the efficient removal of sulfamethoxazole. Sep. Purif. Technol..

[cit90] Wu Y. (2021). *et al.*, Potassium hydroxide-modified algae-based biochar for the removal of sulfamethoxazole: Sorption performance and mechanisms. J. Environ. Manage..

[cit91] Nguyen T.-B. (2022). *et al.*, Mesoporous and adsorption behavior of algal biochar prepared via sequential hydrothermal carbonization and ZnCl_2_ activation. Bioresour. Technol..

[cit92] Zhang T. (2022). *et al.*, Engineering mesoporous algal-based biochars for efficient remediation of norfloxacin pollution in marine environment. Environ. Adv..

[cit93] Ramesh V. (2022). *et al.*, Algal biomass-derived nano-activated carbon for the rapid removal of tetracycline by adsorption: Experimentation and adaptive neuro-fuzzy inference system modeling. Bioresour. Technol. Rep..

[cit94] González-Hourcade M. (2022). *et al.*, Microalgae biomass as a sustainable precursor to produce nitrogen-doped biochar for efficient removal of emerging pollutants from aqueous media. J. Cleaner Prod..

[cit95] Qin J. (2023). *et al.*, Self-activation of potassium/iron citrate-assisted production of porous carbon/porous biochar composites from macroalgae for high-performance sorption of sulfamethoxazole. Bioresour. Technol..

[cit96] Xu S. (2023). *et al.*, Macro- and micro-algae-based carbon composite for pharmaceutical wastewater treatment: Batch adsorption and mechanism study. Process Saf. Environ. Prot..

[cit97] Sun J. (2025). *et al.*, Zeolite-like algal biochar nanoparticles for enhanced antibiotics removal: Sorption mechanisms and theoretical calculations. Colloids Surf., B.

[cit98] Sun J. (2025). *et al.*, Multiple defects algal biochar derived from Ulva lactuca with enhanced adsorption performance for ciprofloxacin. J. Mol. Liq..

[cit99] Kozyatnyk I. (2025). *et al.*, Adsorption of organic contaminants of emerging concern using microalgae-derived hydrochars. Sci. Rep..

[cit100] Nautiyal P., Subramanian K. A., Dastidar M. G. (2016). Adsorptive removal of dye using biochar derived from residual algae after in-situ transesterification: Alternate use of waste of biodiesel industry. J. Environ. Manage..

[cit101] Zhou Y. (2018). *et al.*, Preparation and Characterization of Macroalgae Biochar Nanomaterials with Highly Efficient Adsorption and Photodegradation Ability. Materials.

[cit102] Chen Y.-d. (2018). *et al.*, Highly efficient adsorption of dyes by biochar derived from pigments-extracted macroalgae pyrolyzed at different temperature. Bioresour. Technol..

[cit103] Yao X. (2020). *et al.*, An abundant porous biochar material derived from wakame (Undaria pinnatifida) with high adsorption performance for three organic dyes. Bioresour. Technol..

[cit104] Saeed A. A. (2020). *et al.*, Eucheuma cottonii Seaweed-Based Biochar for Adsorption of Methylene Blue Dye. Sustainability.

[cit105] Gurav R. (2021). *et al.*, Application of macroalgal biomass derived biochar and bioelectrochemical system with Shewanella for the adsorptive removal and biodegradation of toxic azo dye. Chemosphere.

[cit106] Shaikh W. A. (2022). *et al.*, Fabrication of biochar-based hybrid Ag nanocomposite from algal biomass waste for toxic dye-laden wastewater treatment. Chemosphere.

[cit107] Wang Y.-S. (2022). *et al.*, Insights into the highly efficient treatment of dyeing wastewater using algal bloom derived activated carbon with wide-range adaptability to solution pH and temperature. Bioresour. Technol..

[cit108] Kadimpati K. K. (2023). *et al.*, Design of innovative hybrid biochar prepared from marine algae and magnetite: Insights into adsorption performance and mechanism. Chem. Eng. Res. Des..

[cit109] Jafarian S. (2023). *et al.*, Sargassum macro-algae-derived activated bio-char as a sustainable and cost-effective adsorbent for cationic dyes: A joint experimental and DFT study. Colloids Surf., A.

[cit110] Yu H. (2024). *et al.*, Experimental and DFT insights into the adsorption mechanism of methylene blue by alkali-modified corn straw biochar. RSC Adv..

[cit111] Guo S. (2023). *et al.*, Synergistic effect of hydrogen bonding and π-π interaction for enhanced adsorption of rhodamine B from water using corn straw biochar. Environ. Pollut..

[cit112] Spagnuolo D. (2023). *et al.*, Hydrochar from Sargassum muticum: a sustainable approach for high-capacity removal of Rhodamine B dye. RSC Sustainability.

[cit113] Shoaib A. G. M. (2024). *et al.*, Green algae Ulva lactuca-derived biochar-sulfur improves the adsorption of methylene blue from water. Sci. Rep..

[cit114] Chambers C. (2024). *et al.*, Physical and morphological alteration of Sargassum-derived ultraporous superactivated hydrochar with remarkable cationic dye adsorption. Biomass Convers. Biorefin..

[cit115] Zhang H., Zhang F., Huang Q. (2017). Highly effective removal of malachite green from aqueous solution by hydrochar derived from phycocyanin-extracted algal bloom residues through hydrothermal carbonization. RSC Adv..

[cit116] Hellal M. S. (2025). *et al.*, Preparation and characterization of an algal-based magnetic biochar nanocomposite for the removal of azocarmine G2 dye from aqueous solutions. BMC Chem..

[cit117] Soares Dias A. P. (2025). *et al.*, Seaweed-Derived Biochar for Effective Treatment of Dye-Contaminated Wastewater. Water.

[cit118] Badaruddin M. (2025). *et al.*, Study of Selectivity Anionic Dye Removal and Sustainable Regeneration of Hydrochar from Spirogyra sp. Algae Biomass. Trends Sci..

[cit119] Zheng H. (2017). *et al.*, Adsorption of p-nitrophenols (PNP) on microalgal biochar: Analysis of high adsorption capacity and mechanism. Bioresour. Technol..

[cit120] Lu J. (2017). *et al.*, Adsorptive Removal of Bisphenol A Using N-Doped Biochar Made of Ulva prolifera. Water, Air, Soil Pollut..

[cit121] Cui Y. (2019). *et al.*, Phenol and Cr(VI) removal using materials derived from harmful algal bloom biomass: Characterization and performance assessment for a biosorbent, a porous carbon, and Fe/C composites. J. Hazard. Mater..

[cit122] Cheng H. (2020). *et al.*, From macroalgae to porous graphitized nitrogen-doped biochars – Using aquatic biota to treat polycyclic aromatic hydrocarbons-contaminated water. Bioresour. Technol..

[cit123] Zhang C., Lu J., Wu J. (2020). One-step green preparation of magnetic seaweed biochar/sulfidated Fe0 composite with strengthen adsorptive removal of tetrabromobisphenol A through in situ reduction. Bioresour. Technol..

[cit124] Vinayagam R. (2023). *et al.*, 2,4-Dichlorophenoxyacetic acid (2,4-D) adsorptive removal by algal magnetic activated carbon nanocomposite. Chemosphere.

[cit125] Song L. (2024). *et al.*, Oyster shell facilitates the green production of nitrogen-doped porous biochar from macroalgae: a case study for removing atrazine from water. Biochar.

[cit126] Yu C. (2022). *et al.*, Development of a novel biochar/iron oxide composite from green algae for bisphenol-A removal: Adsorption and Fenton-like reaction. Environ. Technol. Innovation.

[cit127] Jagadeesh N., Sundaram B. (2023). Adsorption of Pollutants from Wastewater by Biochar: A Review. J. Hazard. Mater. Adv..

[cit128] Ullah M. H., Rahman M. J. (2024). Adsorptive removal of toxic heavy metals from wastewater using water hyacinth and its biochar: A review. Heliyon.

[cit129] Yin G. (2023). *et al.*, Co-adsorption mechanisms of Cd(II) and As(III) by an Fe-Mn binary oxide biochar in aqueous solution. Chem. Eng. J..

[cit130] Alkhaldi H. (2024). *et al.*, Sustainable polymeric adsorbents for adsorption-based water remediation and pathogen deactivation: a review. RSC Adv..

[cit131] Al-Hazmi G. (2022). *et al.*, Superior Adsorption and removal of doxorubicin from aqueous solution using activated carbon via thermally treated green adsorbent: Isothermal, Kinetic, and Thermodynamic Studies. Environ. Technol..

[cit132] Takeda N. (2024). *et al.*, Solid–liquid partitioning and speciation of Pb(II) and Cd(II) on goethite under high pH conditions, as examined by subnanomolar heavy metal analysis, X-ray absorption spectroscopy, and surface complexation modeling. Chemosphere.

[cit133] Holliday M. C., Parsons D. R., Zein S. H. (2024). Agricultural Pea Waste as a Low-Cost Pollutant Biosorbent for Methylene Blue Removal: Adsorption Kinetics, Isotherm And Thermodynamic Studies. Biomass Convers. Biorefin..

[cit134] Husien S. (2023). *et al.*, Potentials of algae-based activated carbon for the treatment of M.orange in wastewater. Case Stud. Chem. Environ. Eng..

[cit135] Pedrosa M. (2022). *et al.*, Spirulina-based carbon bio-sorbent for the efficient removal of metoprolol, diclofenac and other micropollutants from wastewater. Environ. Nanotechnol., Monit. Manage..

[cit136] Cardoso N. F. (2012). *et al.*, Comparison of Spirulina platensis microalgae and commercial activated carbon as adsorbents for the removal of Reactive Red 120 dye from aqueous effluents. J. Hazard. Mater..

[cit137] Vakili M. (2019). *et al.*, Regeneration of chitosan-based adsorbents used in heavy metal adsorption: A review. Sep. Purif. Technol..

[cit138] Gkika D. A., Mitropoulos A. C., Kyzas G. Z. (2022). Why reuse spent adsorbents? The latest challenges and limitations. Sci. Total Environ..

[cit139] Liu Y. (2024). *et al.*, Green Adsorbents for Environmental Remediation: Synthesis Methods, Ecotoxicity, and Reusability Prospects. Processes.

[cit140] Baskar A. V. (2022). *et al.*, Recovery, regeneration and sustainable management of spent adsorbents from wastewater treatment streams: A review. Sci. Total Environ..

[cit141] Shahid A. (2020). *et al.*, Cultivating microalgae in wastewater for biomass production, pollutant removal, and atmospheric carbon mitigation; a review. Sci. Total Environ..

[cit142] Carvalho J. (2022). *et al.*, Life Cycle Assessment (LCA) of Biochar Production from a Circular Economy Perspective. Processes.

[cit143] Wang S. (2023). *et al.*, Life cycle assessment of carbon-based adsorbent preparation from algal biomass. J. Cleaner Prod..

[cit144] Chen G., Zhao L., Qi Y. (2015). Enhancing the productivity of microalgae cultivated in wastewater toward biofuel production: A critical review. Appl. Energy.

[cit145] Ng I. S. (2017). *et al.*, Recent Developments on Genetic Engineering of Microalgae for Biofuels and Bio-Based Chemicals. Biotechnol. J..

